# Development and Validation of a Modular Sensor-Based System for Gait Analysis and Control in Lower-Limb Exoskeletons

**DOI:** 10.3390/s25082379

**Published:** 2025-04-09

**Authors:** Giorgos Marinou, Ibrahima Kourouma, Katja Mombaur

**Affiliations:** 1 Institute of Computer Engineering (ZITI), Heidelberg University, 69120 Heidelberg, Germany; giorgos.marinou@ziti.uni-heidelberg.de (G.M.); ibrahima.kourouma@stud.uni-heidelberg.de (I.K.); 2 Institute for Anthropomatics and Robotics, Optimization and Biomechanics for Human-Centred Robotics, Karlsruhe Institute of Technology, 76131 Karlsruhe, Germany; 3 Department of Systems Design Engineering, CERC Human-Centred Robotics and Machine Intelligence, University of Waterloo, Waterloo, ON N2L 3G1, Canada

**Keywords:** wearable sensors, wearable robotics, exoskeletons, gait, IMU, 3D-printed insole, force sensors, pressure sensors, crutches, human–robot interaction

## Abstract

With rapid advancements in lower-limb exoskeleton hardware, two key challenges persist: the accurate assessment of user biomechanics and the reliable control of device behavior in real-world settings. This study presents a modular, sensor-based system designed to enhance both biomechanical evaluation and control of lower-limb exoskeletons, leveraging advanced sensor technologies and fuzzy logic. The system addresses the limitations of traditional lab-bound, high-cost methods by integrating inertial measurement units, force-sensitive resistors, and load cells into instrumented crutches and 3D-printed insoles. These components work independently or in unison to capture critical biomechanical metrics, including the anteroposterior center of pressure and crutch ground reaction forces. Data are processed in real time by a central unit using fuzzy logic algorithms to estimate gait phases and support exoskeleton control. Validation experiments with three participants, benchmarked against motion capture and force plate systems, demonstrate the system’s ability to reliably detect gait phases and accurately measure biomechanical parameters. By offering an open-source, cost-effective design, this work contributes to the advancement of wearable robotics and promotes broader innovation and accessibility in exoskeleton research.

## 1. Introduction

Wearable robots, particularly lower-limb exoskeletons (LLEs), have emerged as revolutionary tools in recent years for enhancing mobility in impaired individuals. These devices are critical in managing a broad spectrum of musculoskeletal disorders and injuries, significantly improving the quality of life for affected individuals [1,2,3]. In the vast majority of cases, arm crutches are required in order to operate the exoskeleton [4], and to provide static and dynamic stability through transitions of sitting and standing, as well as walking. LLEs often rely on adjunct technologies, incorporating advanced sensors and control algorithms to closely mimic natural gait patterns. Despite groundbreaking advancements in sensor technology and biomechanical evaluation, the adoption of LLEs faces significant barriers. These challenges include the high costs associated with precise motion capture systems for biomechanical assessment and the complexity of integrating varied technologies into cohesive systems capable of accurately interpreting user intentions in real-time [5,6].

Current methodologies, such as marker-based motion analysis and force plate technologies, though accurate, are predominantly confined to laboratory settings due to their prohibitive costs and extensive setup requirements [7,8]. Recently, marker-less motion capture has begun to address these limitations, offering promising results without the cumbersome setup of traditional systems [9]. However, these technologies still struggle to operate effectively in everyday environments, a critical requirement for practical LLE applications, while still needing camera equipment to cover a certain volume.

The integration of inertial measurement units (IMUs), despite enabling kinematic measurements and gait phase identification outside of lab settings, still faces challenges in calibration and comfort when combined with LLEs [10,11,12,13]. Similarly, surface electromyography (sEMG) quantifies muscular effort but is hindered by preparation requirements and sensitivity to environmental conditions [14,15,16]. Despite these limitations, both IMU and sEMG technologies serve as valuable control input when processed correctly, although unreliability issues due to environmental noise have led to exploring alternative input methods [17,18,19,20,21,22,23,24].

Force-measuring crutches and pressure-sensing insoles equipped with load cells and piezoresistive sensors within force-sensitive resistors (FSRs) offer another way to analyze movement effectively [25,26], at much lower costs (5 to 30 USD per FSR sensor and 250 to 500 USD for industrial-grade load cells). Integrating these with IMUs creates a versatile system that enhances user intention assessment across various platforms. Although these components are proven to effectively analyze gait in multiple studies [25,26,27,28,29,30], there is a notable gap in research combining these technologies into a unified, multi-sensor system. Furthermore, the reproducibility of these methods and hardware is often unclear and complicated, and the cost is relatively high, while lacking open-sourced protocols that hinder further research development. Lastly, while pressure-sensing insoles are commercially available, to the authors’ knowledge, the same does not apply to force-sensing crutches. In the case of commercially available insoles, their applicability in real-time monitoring or integration within a control framework is not possible in the majority of cases, as proprietary restrictions and closed intellectual property rights often limit their adaptability, and their cost ranges between 10,000 to 30,000 USD.

To bridge this gap, our research introduces a cost-effective, easy-to-reproduce, and universally compatible sensor system designed explicitly for LLEs, supporting real-world applications across various terrains [10,14]. Unlike conventional high-cost systems whose precision exceeds practical necessities and often lacks integration capabilities for direct exoskeleton control, our solution prioritizes functional accuracy and ease of integration [31,32]. We have developed a standalone and modular two-part system consisting of crutches equipped with force sensors and insoles that incorporate pressure sensors, both integrated with IMUs. This multi-sensor assembly not only captures synchronized upper- and lower-limb biomechanical metrics in real time but also interprets user intent with high reliability, establishing intuitive control of the exoskeletons. To underscore our commitment to accessibility and community-driven improvement, we provide this technology as an open-source platform, detailed in the Supplementary Materials. To our knowledge, this is the first platform that combines both gait segmentation and upper- and lower-limb biomechanical evaluation in a unified, open-source framework. Table 1 highlights how our system compares to representative insole-based, crutch-based, and combined platforms in terms of sensing, integration, and practical usability.

Moreover, we employ fuzzy logic to process sensor inputs, offering a robust alternative to traditional machine learning methods that require extensive data and often yield unpredictable outcomes. Unlike machine learning, fuzzy logic is computationally inexpensive and follows a rule-based approach, eliminating the need for large datasets that may not exist and avoiding the generation of incorrect data or control inputs for the exoskeleton. This decision-making framework, built on predefined rules, effectively handles the inherent variability in human gait, thus optimizing the exoskeleton’s response to user movements [37,38].

The remainder of this paper is organized as follows: Section 2 details the materials and methods. Section 2.1 introduces the standalone system and the design of its distinct hardware and software components, while Section 2.2 outlines the validation experiment design and analysis. Section 3 presents the results and discussion; Section 3.1 reports the outcomes of the validation experiments and situates them within the current literature, and Section 3.2 explores the system’s potential both as a biomechanical evaluation tool and as a foundation for high-level control architectures in LLEs. Section 4 concludes with a summary of the study’s main implications. A comprehensive guide for replicating the system is also provided in the Supplementary Materials.

## 2. Materials and Methods

**Figure 1:**
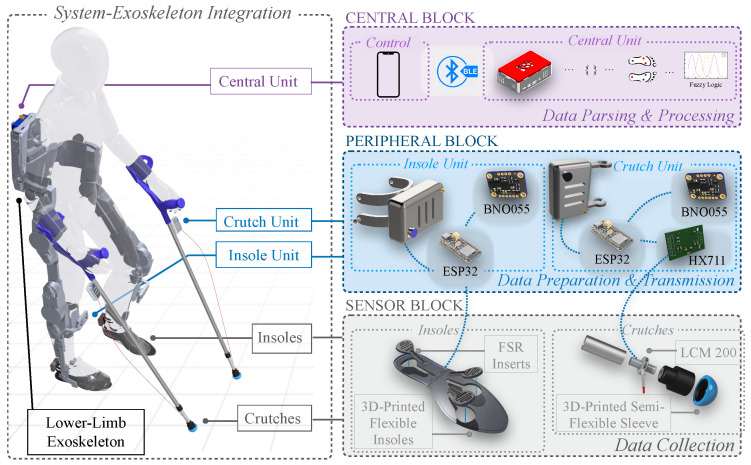
Overview of system components integrated on an LLE. The sensor block includes two flexible 3D-printed insoles and two load cell-instrumented forearm crutches for raw data collection. The peripheral block contains four data collection units (two for insoles, two for crutches) for digitizing and transmitting data. The central block consists of a Raspberry Pi for data parsing and processing and an Android app for system control and operation.

The custom sensor system was designed for collecting biomechanical data with the use of LLEs. Following the development of hardware and software modalities, validation experiments took place in order to assess the accuracy of the sensors and reliability of the system in measuring key biomechanical parameters. This section describes both system development and validation methods. Figure 1 illustrates the system’s integration to LLEs and showcases the three main building blocks of the system. In this study, we test our system using the TWIN LLE [39].

### 2.1. System Description

The modular device consists of two individual sensor systems that can either work together or separately in order to collect biomechanical data from the exoskeleton. A central unit is responsible for handling data collection and wireless communication services between the system components (Figure 1). The first part consists of two instrumented crutches able to measure ground reaction forces (GRFs) using load cells, as well as their acceleration and orientation via IMU units. The second part is a pair of 3D-printed insoles, which are sensorized with three FSRs each, in order to primarily measure the anteroposterior center of pressure (CoP) of the foot, as well as the acceleration and orientation of the foot via an IMU unit. Data from the sensors, processed via ESP32-S3 Feather boards (Adafruit Industries, New York, NY, USA), are transmitted to a central unit using BLE. This unit, powered by a Raspberry Pi 4 (Raspberry Pi Foundation, Cambridge, UK), parses data into a CSV file, while a fuzzy logic algorithm computes gait phases. An Android application manages communication between the central unit and peripherals, overseeing data flow and system operations.

To ensure accessibility and reproducibility, the system was designed with cost-efficiency in mind. The central unit can be assembled for approximately 90.18 EUR. Each insole sensor unit, equipped with three FSRs and one IMU, costs around 110.53 EUR, while each crutch sensor unit, including a load cell and IMU, costs approximately 565.16 EUR. Factoring in the additional materials, connectors, and 3D-printed components, the complete system can be built for under 1500 EUR, which is significantly lower than the cost of commercially available insole systems while offering combined lower- and upper-limb sensing capabilities. A full bill of materials, including a detailed cost breakdown and assembly instructions, is provided in the Supplementary Materials and our online repository [40].

#### 2.1.1. Preliminary Experiments

**Figure 2:**
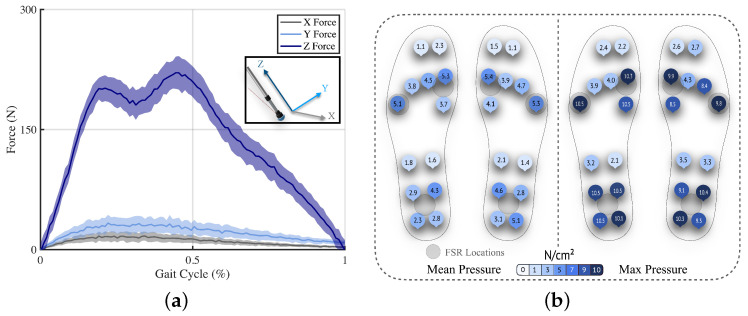
Preliminary experiments for informing system design. (**a**) Comparison of GRF components for exoskeleton-assisted walking. The mean of each component for 30 crutch contact phases is plotted along with the standard deviation, illustrated in the shaded region. (**b**) Pressure distribution during exoskeleton-assisted walking. The average mean (left) and maximum (right) pressures for each sensor are denoted for 30 gait cycles.

Two preliminary experiments informed the design of the system with regards to sensor selection for the crutches and sensor placement for the insoles, and the results are shown in Figure 2. With respect to the crutches, the GRF contributions in the three axes were examined during exoskeleton-assisted gait for one participant, comparing the force vector components from a force plate by translating them into the crutches’ reference systems. A total of 30 crutch contacts were collected by the force plate and averaged over a normalized gait cycle. The transverse components were deemed negligible when compared to the central axis of the crutch, ranging from 12.6 ± 3.74 N for mediolateral forces to 24.2 ± 6.78 N for anteroposterior forces, averaging less than 7% of the vertical forces, as shown in Figure 2a, and agreeing with values previously reported in the literature [35].

For informing the placement of sensors along the insoles, a participant used the Moticon Insoles (Munich, Germany) while walking with the exoskeleton, completing a total of 30 gait cycles. The results were analyzed, showing the highest centers of pressure in the middle of the heel, first, and fifth metatarsal (Figure 2b). In pursuit of simplicity, we positioned three sensors at each of these key locations. This placement aligns with the physical design constraints of LLEs, which typically feature a robust plate at the bottom of the footwear, effectively transforming the foot into a single rigid body driven by ankle flexion. This design assumption supports the division of the foot into two primary contact areas, the heel and the metatarsals, and facilitates a simplified formulation of gait phase estimation outcomes.

#### 2.1.2. Crutches Design

Similar to previous works in literature [25], a pair of standard forearm crutches were cut just above the bottom tip and reinforced with aluminum inner cylinders on both sides, spanning 5 cm long, and featuring threaded inputs. The sensor unit is housed in a 3D-printed case and attached with printed clamps on the shaft of the crutch, just under the handle, which minimally shifts the inertia properties of the crutch. Additionally, a flexible elastic 3D-printed hemispherical sleeve was attached at the bottom tips of the crutches to minimize bending moments about the center of the load cell and help maintain its integrity.

A load cell LCM200 (Miniature threaded in-line, 250 lb, FUTEK, Irvine, CA, USA) [41] was fitted between the two parts, forming a strong connection through the central axis of the crutch (Figure 1). An HX711 analog-to-digital converter (ADC) (Avia Semiconductor, Xiamen, China) [42] was used to read the load cell’s output and relay the data via its 24-bit ADC to the ESP32 unit. The HX711 was chosen due to its low-noise, 24-bit ADC, commonly used for load cells, offering power-down functionality for energy-conscious applications. The BNO055 IMU (Bosch, Reutlingen, Germany) was fitted to collect acceleration and orientation data, while the Adafruit ESP32-S3 Feather MCU (Adafruit, New York, NY, USA) [43] was employed for collecting sensor data, featuring an RGB-LED for visually signaling the state of the peripherals and battery level and a button for direct physical interaction. A rechargeable 3000 mAh, 3.7 V, lithium polymer battery powers each sensor board.

To maintain the simplicity, mobility, low weight and volume, and cost-effectiveness of the crutches assembly, unlike previous studies where strain gauges were integrated along with bulky braces on the sides of the crutch tip [34,36], or tethered to an external computer [44], a uni-axial load cell was chosen based on the preliminary experiments within this study (Figure 2). By exploiting the orientation values from the crutches’ IMUs, we can decompose the resultant GRF into its components and further investigate the anteroposterior and mediolateral components, shedding light on propulsive/braking and balancing forces, respectively. IMU data, including orientation and angular accelerations, and load cell data are serialized to a JSON format. These sensor units conclude the crutches’ peripherals, integrated into the wireless protocol described in Section 2.1.3.

#### 2.1.3. Insoles Design

The design of our 3D-printed insoles is primarily driven by considerations of cost-effectiveness, comfort, and ease of application, such as facilitating sensor exchange among different insole sizes: small, medium, and large (Figure 1). Our main objective is to investigate the anteroposterior CoP, as this metric is particularly valuable for two reasons: (1) it supports gait segmentation by tracking pressure shifts along the length of the foot, enabling accurate identification of gait phases, and (2) it allows for intuitive motion intention detection, as forward shifts in CoP can signal the user’s intention to initiate walking, making it a suitable candidate for triggering exoskeleton assistance. Hence, designs utilizing over 20 sensors to cover the entire foot [26,29,33,45] were deemed excessive. Instead, our design adopts a minimalist approach, strategically placing a reduced number of sensors at critical foot locations. As already shown in previous works [37], gait phases can be detected and categorized using as low as four FSR sensors, but based on the preliminary tests we carried out, we concluded that three sensors would satisfy the requirements of our applications.

The flexible insoles were 3D-printed in small, medium, and large sizes using TPU-90 flexible filament (NinjaTek, Lititz, PA, USA) to ensure a solid yet comfortable surface. The insoles each feature three gaps, where key-shaped inserts containing the FSR (Walfront, Bentonville, AR, USA) sensors can be fitted. The bottoms of the insoles have guided ribbon cables that connect to the inserted sensors. This modular design allows for efficient replacement of sensors should any technical failure occur.

A 3D-printed clamp mechanism allows for the attachment of the sensor cases to the shoe of the exoskeleton. The analog FSR data are converted by the 12-bit ADC onboard the ESP32-S3 Feather. Unlike the industrial-grade load cell used in the crutches, requiring high resolutions for accurately capturing forces, the FSRs used here exhibit significantly higher noise, drift, and hysteresis, typically one to two orders of magnitude greater than load cells [46]. Moreover, the insoles are used to derive relative changes across sensors for estimating center of pressure and gait phase transitions rather than for obtaining absolute force values. Given this context, a 12-bit ADC, such as the one integrated into the ESP32 microcontroller, is sufficient for capturing relative pressure distributions without introducing unnecessary system complexity. To this end, no calibration of the FSRs prior to use is necessary. Instead, all algorithms, such as those estimating the center of pressure or gait phase, use the relative distribution of sensor values. This approach minimizes the impact of sensor drift or variability across participants. A brief warm-up period was used at the beginning of each session to allow the FSRs to stabilize. A BNO055 IMU is also included in the sensor unit. For all IMUs, a short calibration procedure is necessary prior to every experiment. Angular accelerations and orientations from IMUs, along with FSR readings, are serialized to a JSON format. These sensor units complete the insole peripherals integrated into the wireless protocol described in the following subsection.

#### 2.1.4. Central Unit and Data Handling

The central unit consists of a Raspberry Pi 4 Model B, ensuring seamless communication and computation across the four sensor peripherals. It is powered by a battery pack mounted on a custom 3D-printed interface designed to fit most standard LLEs. Wireless communication is established via Bluetooth 4.0, enabling high-speed, energy-efficient data transmission using both Bluetooth Low Energy (BLE) and Bluetooth Classic protocols, depending on the required throughput.

Sensor units transmit serialized JSON data packets to the central unit, which are then deserialized and saved in a tabular CSV format. These data streams are simultaneously fed into the fuzzy logic algorithm for real-time processing. The system supports both insole and crutch subsystems independently or in combination, depending on the intended application. An RGB LED connected to the Raspberry Pi provides visual feedback for key events such as sensor connection, recording start/end, and power status.

The final recorded dataset includes three-dimensional angular accelerations and orientations from the four IMUs, amplified load cell outputs from the crutches, and FSR readings from both insoles, synchronized at each timestamp. To improve communication efficiency, floating-point values were limited to four decimal places. The data acquisition frequency can be adjusted to optimize throughput or range, depending on whether the central unit is mounted on the user or located remotely. Wireless data transmission remains stable up to 30 m at a maximum frequency of 130 Hz.

System control is facilitated through a dedicated Android application, allowing users to calibrate the system, initiate or end recordings, manage sensor selection, and trigger external devices such as camera systems via an accessory module. Data recording with active Bluetooth communication between peripherals and the central unit can be maintained for up to 12 h, ensuring reliability during full-session use. The system’s durability has been validated over a three-hour active use window with no decline in sensor performance. Full details on integration and custom device support are available in the Supplementary Instruction Manual.

#### 2.1.5. Technical Considerations

To ensure robustness against environmental noise and sensor drift, several processing steps are applied to the raw sensor signals. For the IMUs, raw accelerometer and gyroscope outputs are first passed through internal low-pass filters using the BNO055’s default configuration (accelerometer bandwidth: 62.5 Hz; gyroscope bandwidth: 32 Hz). These filtered signals are then fused via Bosch’s proprietary sensor fusion algorithm, which internally relies on a Kalman filter to compute robust orientation estimates from the IMU’s triaxial accelerometer, gyroscope, and magnetometer readings [47]. For the FSR sensors, absolute signal values are not used in isolation. Instead, all computations, including CoP estimation and fuzzy logic-based gait phase detection, rely on the relative values between sensors. This design choice enhances robustness by minimizing the impact of noise, drift, or absolute calibration deviations.

Operating in a multi-IMU environment presents inherent synchronization challenges, particularly due to independent real-time clocks (RTCs) and potential drift over time [48,49,50]. To overcome this limitation, we adopted a centralized oversampling strategy at the master (Raspberry Pi) level: all incoming data packets from the IMUs and sensors are timestamped upon arrival by the master system. This eliminates the need to rely on peripheral RTCs and enables a shared synchronized timeline across sensors. Although this method does not provide absolute sampling timestamps, it mitigates clock drift artifacts while maintaining flexibility in sensor selection and reducing latency compared to traditional time-synchronization protocols.

To safeguard against sensor malfunctions or communication dropouts, the data collection unit implements an adjustable timeout window during which data packets are expected from all sensor peripherals. Only when all required data are received within this window are the signals processed further to potential external sources for control. If data from any sensor are missing or delayed beyond the timeout threshold, the data stream is marked as incomplete, and the safety logic prevents further processing or control actions based on this information.

#### 2.1.6. Fuzzy Logic

Fuzzy logic was employed to process FSR values collected from the insoles to effectively estimate the gait phase. Commonly found works [45,51] utilize membership functions expressed as sigmoid functions that evaluate the absolute values of FSR readings,

(1) $$\mu \left(F_{i}\right)=\frac{1}{1+e^{-s*(F_{i}-F_{0})}}$$

with $F_{i}$ being the FSR readings and $\mu (F_{i})$ representing the membership grade as a result of an FSR reading being evaluated by the membership function. The parameter *s* determines the slope of the sigmoid curve and $F_{0}$ defines the threshold in terms of the absolute value, meaning the point at which $F_{1}$ yields a membership grade of 0.5. An option is to utilize half of the maximum possible FSR reading for $F_{0}$. The greater the *s*, the greater the change in $\mu (F_{i})$ with small changes in $F_{i}$.

During testing with the fuzzy logic algorithm, it was observed that the FSR readings often did not achieve their theoretical maximum values, even under loads exceeding their specified ratings. Consequently, this approach of formulating membership functions may lead to less accurate results that hinder the accuracy of phase estimation. To mitigate the effects of saturation in FSRs, the readings should be normalized for primarily taking into account different walking styles and weight distributions [45,51], by scaling down each FSR reading with the sum of all readings,

(2) $$F_{i}^{*}=\frac{F_{i}}{\sum_{i=1}^{n}Fi}.$$

This normalization of the FSR values not only mitigates the effects of sensor saturation but also enhances the generalizability of the fuzzy logic rules across different users. By scaling each FSR reading relative to the total value, the system adapts to variations in user weight distribution, walking styles, and gait abnormalities without the need for user-specific calibration. Furthermore, the sum of the membership grades across all gait phases provides an internal consistency check: values significantly below 1 may indicate gait abnormality, while values above 1 suggest conflicting input patterns that prevent classification into a distinct phase. This mechanism enables the system to tolerate variability and detect irregularities while maintaining reliable performance across users with different biomechanical profiles. Hence, a membership function is used

(3) $$\mu \left(F_{i}\right)=\frac{1}{1+e^{-0.15*(F_{i}-0.45)}}$$

to express the linguistic variables ‘High’ and ‘Low’ following the work of Kong and Tomizuka [37], which are utilized for the fuzzy representation of FSR readings, where

(4) $$\begin{array}{cc} \mathrm{High} & =\mu (F_{i})\\ \mathrm{Low} & =1-\mu (F_{i}). \end{array}$$

**Figure 3:**
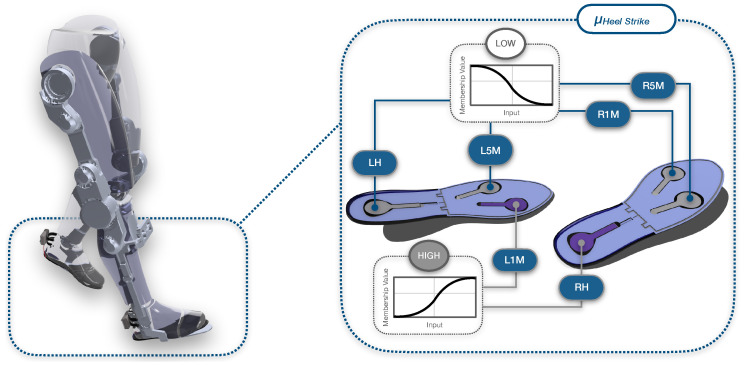
Example of heel strike through fuzzy logic rule-based approach. During exoskeleton gait, the gait phase is decided through a set of rules based on the membership grades assigned to the FSRs of both insoles using membership functions. Linguistic variables and logical expressions are used in order to decide the outcome, or gait phase.

Figure 3 demonstrates an example case of these linguistic variables, used to construct the fuzzy rules that are based on fundamental logical expressions in the form of

(5) $$\begin{array}{cc} \; & \mathrm{IF}\;F_{\mathrm{LH}}^{*}\;\mathrm{is}\;\mathrm{LOW},\;\mathrm{AND}\;\mathrm{IF}\;F_{L5M}^{*},F_{L1M}^{*},F_{\mathrm{RH}}^{*}\;\mathrm{are}\;\mathrm{HIGH},\\ \; & \mathrm{AND}\;\mathrm{IF}\;F_{R5M}^{*},F_{R1M}^{*}\;\mathrm{are}\;\mathrm{LOW},\;\mathrm{THEN}\;G\;\mathrm{is}\;\mathrm{HEELSTRIKE} \end{array}$$

where $F^{*}$ designated with the respective subscript represents the scaled-down FSR reading of each sensor placement, while G represents the gait phase.

The abbreviations LH,L1M,L5M,RH,R1M,R5M stand for the heel, first metatarsal and fifth metatarsal of the left and right foot, respectively. Conditional statements, such as $\mathrm{IF}\;F_{\mathrm{LEFT}\;\mathrm{HEEL}}^{*}\;\mathrm{IS}\;\mathrm{HIGH}$ determine the membership grade of $F^{*}$ to a particular fuzzy set or linguistic variable, which, in this instance, is HIGH. Chaining several of these expressions with the AND operator yields the minimum membership grade, representing the membership grade of G to a particular gait phase. This is expressed by conclusive statements like THENGISHEELSTRIKE, illustrated in Figure 3 where the two ‘high’ sensors are in a darker colour and the rest ‘low’ sensors in a lighter shade, during a heel strike phase.

Table 2 presents all rules for gait phase estimation with respect to the right foot. To obtain the rules for the left side, the conditional statements in each rule need to be inverted. The presence of red circles signifies the condition $F^{*}\;\mathrm{IS}\;\mathrm{HIGH}\;$ for an FSR placement. Once all rules are evaluated, each of the eight gait phases is assigned a membership grade.

### 2.2. Validation Experiments

**Figure 4:**
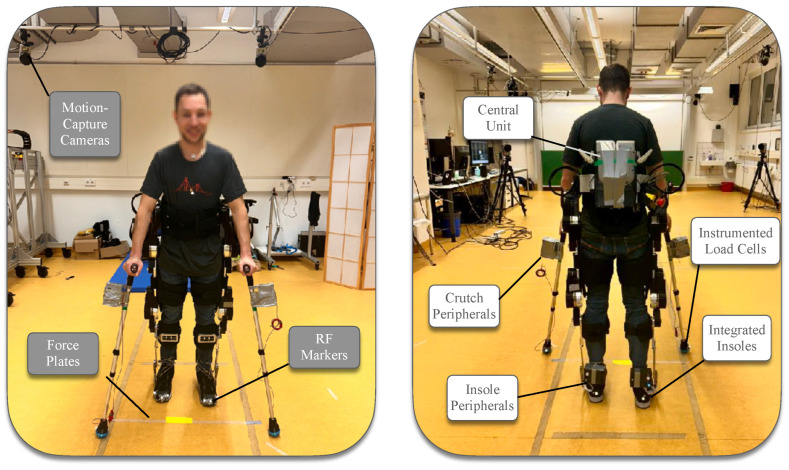
Experimental setup using the crutches and insoles system. The two sub-systems, the central unit is securely mounted on the onboard computer interface of the exoskeleton in the back. Gold standard equipment methodologies used to validate the system are indicated.

The experiments carried out within this study aimed to validate the technical accuracy and reliability of the proposed sensor-based system in capturing key biomechanical metrics: anteroposterior center of pressure, crutch ground reaction forces, and heel strike detection. Three different experiments took place in order to validate the hardware and software capabilities of the system against the gold standard marker motion capture (Qualisys, Gothenburg, Sweden) and force plate (Kistler Group, Winterhur, Switzerland) technologies. The first two experiments comprised of standalone equipment testing, while for the third experiment, three able-bodied participants (one female and two males, age 36 ± 7 years, weight 68.7 ± 12.1 kg, height 1.67 ± 0.08 m) were recruited. The participants performed a set of six walking bouts each, with the TWIN lower-limb exoskeleton [39]. All participants signed informed consent forms prior to the experiments, the procedures of which were in accordance with the Declaration of Helsinki and approved by the Ethical Committee of Heidelberg University (resolution S-313/2020). Figure 4 demonstrates the integration of the system to the LLE TWIN and the equipment used for the validation experiments.

All post-processing computations, data and statistical analyses were conducted using MATLAB R2023b (MathWorks Inc., Natick, MA, USA). For the synchronization of data recordings, a trigger sub-system was created using an ESP32 Firebeetle, which was connected to the Qualisys system, incorporating the force plates, and sent a trigger signal through at the start of every recording.

#### 2.2.1. Anteroposterior Center of Pressure

The center of pressure is a critical metric for assessing balance and understanding the intentions—whether propulsive, braking, or stationary—of an exoskeleton user. For estimating gait phases and detecting user motion-intention, the anteroposterior direction (the direction of travel) plays the most critical role. Consequently, the anteroposterior CoP, measured along the length of the foot, was prioritized in our analysis of the system’s capabilities. To capture the entire area of the feet, a person placed one foot on a force plate and performed circular clockwise motions, shifting their center of mass to record comprehensive CoP data. This circular motion was performed 18 times across three separate experiments. The CoP was then calculated from the FSR data of the insoles by computing the weighted averages for mediolateral and anteroposterior coordinates using the locations of the FSRs,

(6) $$y_{\mathrm{CoP}}=\frac{\left(y_{H}\cdot F_{H}\right)+\left(y_{M}1\cdot F_{M}1\right)+\left(y_{M}5\cdot F_{M}5\right)}{F_{\mathrm{total}}}$$

where $y_{H},y_{M}1,y_{M}5$ are the anteroposterior coordinates of the heel, first metatarsal, and fifth metatarsal sensors, respectively; $F_{H},F_{M}1,F_{M}5$ are the FSR values measured at these points and $F_{\mathrm{total}}$ is the sum of these values. Subsequently, the force plate CoP was transformed using the markers on the shoe from the global laboratory reference system to the insole reference system, as to enable comparison between the two values.

#### 2.2.2. Crutches Ground Reaction Forces

A crucial yet often overlooked metric in the assessment of exoskeleton-assisted gait is the contribution of the upper body. To address this gap, our instrumented crutches are designed to provide detailed insights into how users utilize forearm crutches to support their gait. In one of the experiments, a person repeated a total of 18 circular movements over three experiments while applying forces to the crutch and maintaining it in contact with a force plate. The resulting force vector, captured by the force plate, was then translated into the crutch’s reference system using markers placed on the crutch. The data from the load cells were subsequently compared to the translated vertical component of the force plate, which aligned with the crutch’s central axis.

#### 2.2.3. Heel Strike Gait Detection

To assess the capability of our sensors to accurately estimate gait phases, we focused on a fundamental variable: the heel strike. Three participants, each equipped with the system and markers, performed 18 trials across the laboratory using the exoskeleton. By analyzing the data from the heel FSRs, we identified the time frames of local maxima, which were then compared to those captured by motion capture cameras. Heel strikes from markers were defined by calculating the maximum and minimum distances between toe and sternum and heel and sternum markers, as outlined in our prior research [5]. Toe-off events were also analyzed to provide further comparative data and enhance the evaluation of our system’s robustness.

#### 2.2.4. Data and Statistical Analysis

Data were collected from our custom system, motion capture cameras, and force plates at sampling rates of 130 Hz, 150 Hz, and 1500 Hz, respectively. To ensure clarity and remove noise, a Butterworth filter with respective orders and cut-off frequencies was applied to each data stream separately. For comparative analysis, the data from higher sampling rates were downsampled to match the lowest frequency data, thus normalizing the datasets. This approach to data treatment preserved the integrity and essential features of the raw data, ensuring an accurate representation for further analysis.

For our statistical analysis, a Shapiro–Wilk test at a significance level of α=0.05 revealed a non-normally distributed nature for our datasets. This led to all of our comparisons between paired datasets of biomechanical metrics collected using two different systems to be made using the non-parametric Wilcoxon Signed-Rank test, where p<0.05. Hence, our data are reported as median and inter-quartile range (IQR) and based on the significance of the results, we report the median and IQR values for the differences between different collection methods, and the minimum, maximum, mean and standard deviations (STD) of root-mean-square error (RMSE) and the Pearson correlation coefficients between the datasets. Results are reported for the left side insole and crutch, as the Wilcoxon Signed-Rank test between the two sides did not show a significant difference.

## 3. Results and Discussion

Lower-limb exoskeletons have advanced wearable technology by significantly restoring mobility and rehabilitating impaired gait for individuals with movement disorders [52]. However, their efficient integration into daily life requires a thorough biomechanical assessment, typically reliant on costly lab-based equipment that fails to capture critical biomechanical parameters in practical settings. Moreover, achieving transparent control remains challenging, with current solutions often depending on expensive sensors or complex machine learning techniques that may lack adequate user feedback and comfort.

To address these challenges in a cost-effective and user-centered manner, we developed a modular sensor-based system for typical LLEs, capable of recording key biomechanical metrics for assessment and motion intention-based control. The system includes forearm crutches with load cells and flexible 3D-printed insoles with pressure sensors, both integrated with IMUs. Data are processed through a fuzzy logic algorithm for efficient and accurate gait phase estimation. Additionally, we provide full open-source access to our hardware and software designs, along with instruction manuals. This approach advances real-life applications of LLEs, and to the authors’ knowledge, these technologies have not been combined before, particularly in such a low-cost, simple, and effective manner.

### 3.1. Results of Validation Experiments

To validate our system and the accuracy of the data recorded from the sensors, we performed a set of validation experiments against force plate and motion capture systems, based on three distinct metrics: (1) the anteroposterior center of pressure as recorded from the insoles, (2) the ground reaction forces as recorded from the crutches, and (3) the timestamp of successive heel strikes as reported from the system’s software. It is important to note that the goal of this study was not to generalize biomechanical behavior across a population but rather to validate the technical accuracy and reliability of the proposed sensor-based system in capturing these key biomechanical metrics. Hence, the selected validation experiments were designed to demonstrate the system’s capability under controlled conditions rather than to assess inter-subject variability.

**Figure 5:**
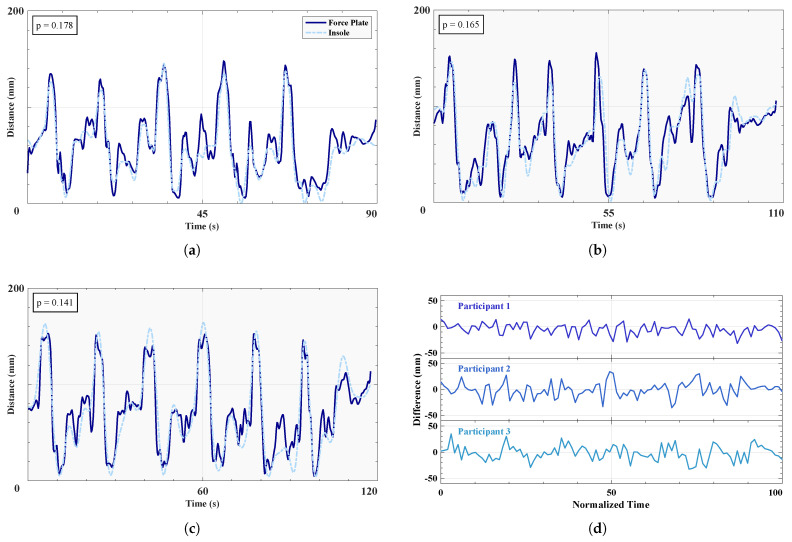
Anteroposterior center of pressure comparison between insole and force plate. (**a**–**c**) Three time series of the anteroposterior CoP as recorded by force plate and insole, from each participant in order, performing circular motions about their foot. *p* values are provided for each trial. (**d**) Differences in anterposterior CoP between the force plate and the insole, over all three trials.

**Figure 6:**
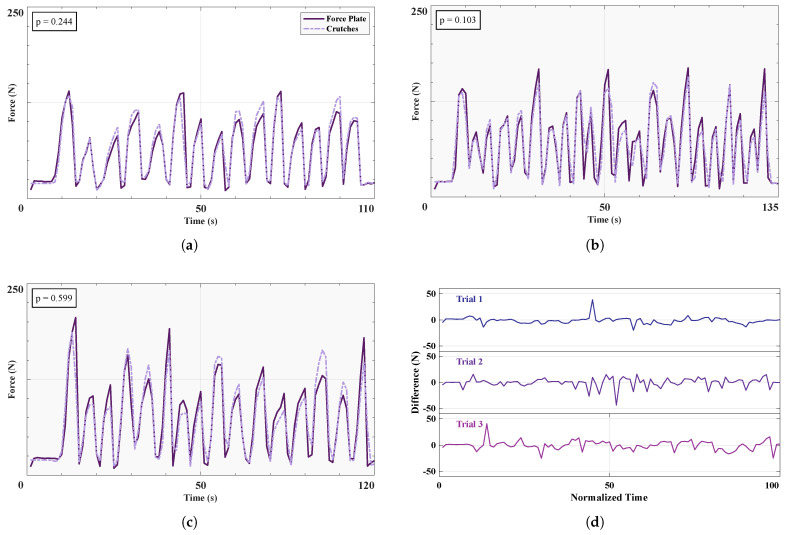
Ground reaction forces comparison between crutch and force plate. (**a**–**c**) Three time series of the GRFs as recorded by force plate and insole, from each participant in order, performing circular motions about their foot. *p* values are provided for each trial. (**d**) Differences in GRFs between the force plate and the insole, over all three trials.

**Figure 7:**
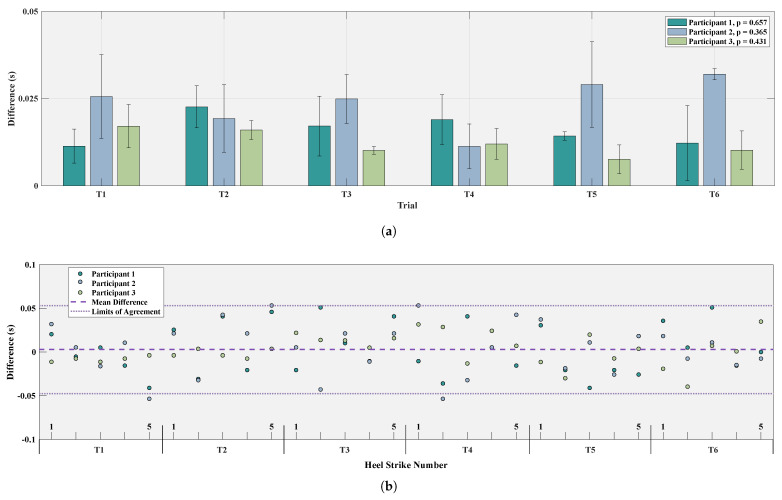
Heel strike comparison between insoles and markers. (**a**) Bar plots for six trials reporting the median and inter-quartile ranges of the differences in heel strikes for each of the three participants. *p* values reported in the legend for each participant. (**b**) Difference of markers and insole heel strikes plotted against each participant heel strike over six trials, along with the median difference and limits of agreement.

Figure 5, Figure 6 and Figure 7 visualize the comparative performance of our system against these gold-standard approaches, and Table 3 summarizes the key primary statistical outcomes extracted from the results, reporting on the accuracy of the three metrics. For the anteroposterior CoP and the crutches GRF, mean and standard deviation values are reported for the Pearson correlation ratio and the RMSE, as calculated over three independent trials performed by a single participant. For the heel strike gait detection, these values are calculated across three participants, each performing six walking bouts.

#### 3.1.1. Anteroposterior Center of Pressure

The plantar CoP is a crucial metric for both assessing balance and informing the control of LLEs, with the AP component indicating whether the user intends to move forward or remain stationary. The AP coordinates of the CoP as recorded from the force plate and the insoles are shown in Figure 5, parts A to C, for each of the three trials separately, as well as the actual difference values in part D, for each trial separately. Table 3 shows that the mean RMSE of the difference between the left insole and the force plate measurements over all three trials is 17.2 ± 2.50 mm. This indicates a high degree of accuracy, suggesting that the insole measurements closely match those of the force plate. The *p*-values for the first, second, and third trials are 0.178, 0.165, and 0.141, respectively, indicating no statistically significant difference between the insole and force plate measurements. A high Pearson coefficient of 0.907±0.038 indicates a close correlation between the two measurement technologies.

The anteroposterior center of pressure measured by our insoles demonstrates a high degree of accuracy when compared to force-plate generated CoP data, comparable to previous research [53] (Pearson correlation coefficients 0.84–0.90). For analyzing gait intentions and determining whether specific sensor membership grades within our fuzzy logic algorithm vary from high to low, our system is more than adequate, as shown by the close matches of high and low peaks in Figure 5. Additionally, the high level of accuracy provided by the insoles offers a reliable biomechanical metric for assessing balance in assisted gait, achieved through a minimal array of three sensors. In terms of informing the control of LLEs, it can be inferred that the system is deemed highly robust in translating the intention of the user to move, since a forward shift in the sagittal plane, or direction of travel, will be captured most accurately.

#### 3.1.2. Crutches Ground Reaction Forces

Upper body contributions in exoskeleton-assisted gait are often overlooked, thus, the effort exerted by the user cannot truly be quantified as a whole. For long-term use of assistive exoskeletons, upper body efforts need to be evaluated and minimized to achieve an energy-efficient integration of exoskeletons into daily life [54]. Forearm crutches with GRFs provide important insight into how much upper body effort users exert when using LLEs. Figure 6a–c reports the GRFs recorded from the left crutch for each of the three trials separately, while Figure 6d displays the median and IQR of the differences between the two collection systems over the three trials. The values for the Pearson coefficients (0.945±0.023) and RMSEs (15.3±4.21) are reported in Table 3. The mean RMSE for the force plate and crutch is 15.3±4.21 N over three trials, and the *p* values range from 0.103 to 0.599.

The GRFs recorded from the instrumented crutches achieved a very high level of accuracy when compared to values obtained from force plates, as shown both in Figure 6 and Table 3. The deviation of the crutches was minimal, and the percentage mean RMSE of the ground-truth GRF (4.84%) is comparable to similar instrumented crutches studies using more comprehensive sensor arrays [34,35] (2–5.4% mean RMSE of total crutch GRF). In conclusion, our instrumented crutches offer a cost-effective and reliable method for accurately investigating upper body contributions via GRFs in LLE-assisted gait.

#### 3.1.3. Heel Strike Gait Detection

Whether assessing key biomechanical metrics such as gait speed and stride duration or accurately segmenting gait cycles from beginning to end, the heel strike is arguably the most important phase indicator [55]. The heel strike timestamps detected from markers on the left shoe and the FSRs of the left insole are compared in Figure 7. Part A reports the median and IQR values for each participant and each of the six trials separately, whereas part B visualizes the errors for each individual heel strike over the three participants, as compared to the heel strikes calculated from marker data.

The mean RMSE between the markers and the insole over the six trials for all three participants is 0.0291±0.0084 s, as reported in Table 3, highlighting the insole’s high precision and robustness. This corresponds to a mean absolute error of 28.1 ms, which indicates high precision and performance of the system, especially in the context of exoskeleton gait. When compared to marker-derived timestamps of heel strikes, our method demonstrated a mean error of 0.844% ± 0.317% relative to the respective step durations, where the mean step duration with the exoskeleton was 3.33 ± 1.16 s.

Figure 7b shows close agreement between marker-calculated heel strikes and insole, supported by a very strong Pearson correlation of 0.998±0.001 across all participants. The limits of agreement present on the figure correspond to ±1.96 SD (95% confidence), and the equal spread of data below and above the mean highlight that the errors were symmetrically distributed around the true values, indicating no systematic bias in the method. Comparable to previous studies using insoles to identify heel strikes and segment gait, with a mean absolute error of 0.0168 s ([56,57] mean absolute errors 0.01 to 0.03 s), our insoles demonstrate high precision, which validates the calculation of gait phase duration, as well as the use of FSR sensors within the fuzzy logic context for a rule-based approach to gait phase estimation.

It is important to note that while the sample size in this study is relatively small, it is sufficient for assessing the accuracy of the sensor system in capturing specific biomechanical metrics. However, to more comprehensively evaluate the system’s robustness and reliability across varied conditions and users, additional testing with a larger and more diverse participant pool is necessary. A broader behavioral evaluation has been conducted in a separate outdoor study, which is currently under preparation and will further build on the findings presented here. Additionally, of utmost importance is the implementation of the proposed control strategies (see Section 3.2) with an LLE and the validation of the system’s efficacy in real-time use of exoskeletons. Future studies will focus on implementing these controllers and refining software control strategies based on the preliminary outcomes presented in this study.

Qualitative feedback from users during laboratory trials and early field experiments indicated that the system did not interfere with the natural use of the exoskeleton. Participants reported that the 3D-printed insoles were comfortable to walk with, and the pressure sensors were not perceptible during gait. The modified crutches preserved the original ergonomics: the sensor unit was placed directly below the handle to minimize any change in the crutch’s moment of inertia, and the elastic tip modification did not alter user perception or crutch-ground interaction. No discomfort or usability concerns were reported, supporting the system’s viability for extended use.

This research established a comprehensive validation of our prototyped system to gather real-time biomechanical data and inform the gait analysis and control of exoskeletons. We have demonstrated a very strong correlation of basic biomechanical metrics calculated using our system compared to the gold-standard force plate and motion capture systems’ ground truths. Our results are comparable, if not superior, to current alternatives, offering distinct advantages of low production costs and design simplicity. Additionally, all of our work is provided open-source to ensure reproducibility and foster collaborative improvements in future iterations.

### 3.2. Discussion on Future Applications

Our modular sensor system offers a versatile platform for advancing the functionality and assessment of LLEs. This section explores the two primary applications of our system: biomechanical evaluation and high-level control. By leveraging a comprehensive array of biomechanical metrics and sophisticated control algorithms, our system not only offers the potential to enhance the understanding of exoskeleton-user interactions but also significantly contributes to the development of responsive and adaptive assistive technologies.

#### 3.2.1. Biomechanical Evaluation

The integration of IMUs and load cells in the crutches captures upper-body dynamics such as propulsive and stabilizing GRFs, while the FSRs in the insoles enable the continuous tracking of CoP trajectories. Together, these modalities provide rich biomechanical metrics across both the upper and lower limbs, including stride duration, gait velocity, and limb orientation, derived in real time. By fusing data from both crutches and insoles, the system enables robust gait phase detection, aligning exoskeleton support with natural user movement. Notably, the system’s lightweight and modular design allows for deployment outside the lab, supporting field-based evaluations. Finally, the open-source and adaptable architecture would potentially allow researchers to tailor the system to specific study needs or clinical applications, promoting broader accessibility and innovation.

#### 3.2.2. High-Level Controller

**Figure 8:**
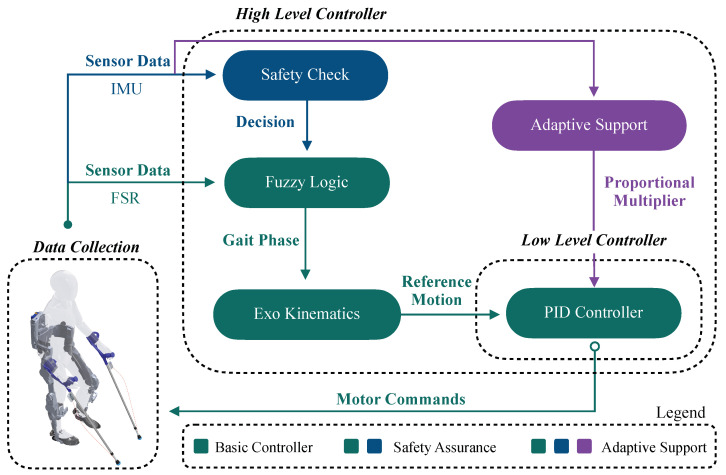
Proposed controllers example. The green schematic describes the normal processes of the basic controllers, whereas the additional blue controls outline the extra functions incorporated by the safety assurance controller within the higher level controller of the system. The purple schematics introduce the proportional multiplier directly applied onto the PID controller of the lower level control of the exoskeleton.

Our modular sensor system enables the extraction of biomechanical variables that are suitable for integration into real-time exoskeleton control strategies. Although we do not implement a complete closed-loop control architecture in this work, we demonstrate the feasibility of doing so by proposing three feedback-based control schemes derived from the sensor data. These schemes, described in detail in Figure 8, showcase how different subsystem configurations (e.g., insoles, crutches, or both) could be leveraged to achieve motion intention detection and responsive assistance through fuzzy logic frameworks.

- Basic Gait Phase Detection Controller

A basic control loop utilizes a simple fuzzy logic approach based on FSR voltages to identify distinct gait phases. The controller processes the sensor data to construct membership functions that determine the current phase of the gait cycle. Once a specific gait phase is recognized, the controller sends a signal to the exoskeleton to initiate or adjust movement, ensuring that the device is synchronized with the user’s natural walking pattern. This basic control loop is crucial for smooth operation and enhances the natural feel of the exoskeleton during use.

- Safety Assurance Controller

Building on the basic gait phase detection, a safety assurance controller could be developed to incorporate additional logic layers that verify the orientation and stability of both crutches and insoles. In this conceptual design, the system would confirm the ground contact of both crutches, detect forward foot motion via IMUs, and check orientation limits to prevent unstable or unsafe actions. Should any sensor input be missing or deemed unreliable—identified, for example, by a timeout mechanism at the central unit—the system could block movement commands and revert the exoskeleton to a predefined safe posture. The state model used in this framework comprises three key postures: Initial Position, Left Strike, and Right Strike. Transitions would only occur if phase progressions are physiologically plausible (e.g., preventing a heel strike immediately after a loading response). These safety checks are proposed as fail-safes for future implementations, ensuring movement only occurs under safe, validated conditions.

- Adaptive Support Controller

The most advanced conceptual control loop we propose is the adaptive support controller, which, if implemented, would not only recognize gait phases and ensure safety but also adjust the level of support provided by the exoskeleton in real time. This approach would use GRF data from crutches to modulate the support intensity—higher forces could trigger increased assistance, adapting to the user’s exertion level. IMU data could be used to estimate the inclination of the crutches, which may correlate with walking speed intentions. For instance, a lower inclination angle might indicate a desire to walk faster, prompting the controller to adjust the response time of support mechanisms accordingly. This proportional control concept is envisioned to provide exoskeleton assistance that is responsive and personalized, dynamically adjusting to changes in walking dynamics or user fatigue.

## 4. Conclusions

This study addressed the challenges of cost-effective and user-centered integration of LLEs into daily life by developing a modular sensor-based system capable of recording key biomechanical metrics for both assessment and motion intention-based control. The system, comprising force-sensing forearm crutches and 3D-printed insoles with integrated IMUs and FSRs, utilizes a fuzzy logic algorithm for efficient gait phase estimation. Validation experiments against force plate and motion capture systems demonstrated high accuracy in measuring the anteroposterior center of pressure, ground reaction forces, and heel strike detection. Our results, which showed strong correlations and low RMSE values, confirmed that the system effectively captures critical biomechanical parameters. The open-source availability of our hardware and software designs further promotes innovation and broader application in exoskeleton research. By meeting its objectives, this study significantly advances the practical usability and assessment of LLEs, fostering further development in wearable robotics and assistive technology.

## Figures and Tables

**Table 1:** Comparison of research-based instrumented insole and crutch systems for biomechanical data collection. GS: Gait Segmentation, CoP: Center of Pressure, GRFs: Ground Reaction Forces. ^1^ System can only measure GRFs from crutches, not insoles.

Study	Sensors & Hardware (per Side)	Biomechanical Outcomes			Wireless Communication	Exoskeleton Integration	Open Source
		GS	CoP	GRFs			
**Insoles**							
González et al. (2015) [29]	4 FSRs, 1 IMU	Yes	No	No	Yes	No	No
Khandakar et al. (2022) [26]	16 FSRs	Yes	No	Yes	Yes	No	No
Senanayake et al. (2010) [33]	4 FSRs, 1 IMU	Yes	No	No	No	No	No
**Crutches**							
Sardini et al. (2015) [34]	6 Strain Gauges	No	No	Yes	Yes	No	No
Seylan et al. (2018) [35]	4 FSRs, 1 Accelerometer	No	No	Yes	No	No	No
Chen et al. (2018) [36]	1 Load Cell, 4 Strain Gauges	No	No	Yes	Yes	Yes	No
**Combined Systems**							
Marinou et al. (this work)	3 FSRs, 1 Load Cell, 2 IMUs	Yes	Yes	Yes ^1^	Yes	Yes	Yes

**Table 2:** Fuzzy logic rules and gait phase outcomes based on FSR insole membership grades. Outcomes based on right insole.

	FSR Grades ($F^{*}$)						Outcome
	LH	L5M	L1M	RH	R5M	R1M	
Value	Low	High	High	High	Low	High	Heel Strike
	Low	Low	High	High	High	Low	Loading Response
	Low	Low	Low	High	High	High	Mid-stance
	High	Low	Low	Low	High	High	Terminal Stance
	High	Low	Low	Low	Low	High	Pre-swing
	High	High	High	Low	High	Low	Initial Swing
	High	High	High	Low	Low	Low	Mid-Swing
	Low	High	High	Low	Low	Low	Terminal Swing

**Table 3:** Pearson Correlation Coefficients and RMSE. The mean and standard deviation is reported across three repeated trials for anteroposterior center of pressure and crutch ground reaction forces, and across three participants performing six walking bouts each for heel strike detection.

	Anteroposterior Center of Pressure		Crutch Ground Reaction Forces		Heel Strike Gait Detection	
	Pearson Corr.	RMSE (mm)	Pearson Corr.	RMSE (N)	Pearson Corr.	RMSE (s)
Min	0.873	14.4	0.921	10.5	0.997	0.0195
Max	0.948	19.2	0.967	17.9	0.999	0.0362
Mean	0.907 ± 0.038	17.2 ± 2.49	0.945 ± 0.023	15.3 ± 4.21	0.998 ± 0.001	0.0291 ± 0.0084

## Data Availability

The raw data supporting the conclusions of this article will be made available by the authors on request.

## Supplementary Materials

The following supporting information can be downloaded at: https://www.mdpi.com/article/10.3390/s25082379/s1, Figure S1: Common components of Insole and Crutch Units. Figure S2: Additional components for Crutch Unit. Figure S3: Breadboard layouts. Figure S4: Front and back of the breadboard with soldered components. Figure S5: Cable for connection with LED. Figure S6: Cable for connection with button. Figure S7: Load cell wires screwed into screw terminal. Figure S8: Plugged-in ESP32, HX711 and LED and button cables. Figure S9: Connected battery and STEMMA QT cable. Figure S10: Additional components for Insole Unit. Figure S11: Breadboard connection layout. Same color coding as in Figure S3 with the addition that grey lines represent the resistors. Figure S12: Front and back of the breadboard with soldered components. Figure S13: First part of the three-part cable system. Figure S14: Second part of three-part cable system. Figure S15: Third part of three-part cable system. Figure S16: Plugged-in board. Figure S17: Spacer sleeves package with M3 form factor. Figure S18: Breadboard with fixated sleeves on all four corners. Figure S19: Horizontally aligned breadboards. Figure S20: Complete Crutch Unit board. Figure S21: Components for external trigger. Figure S22: Complete External Trigger Unit board. Figure S23: Insole joints. Figure S24: BLE scanner app. Figure S25: Raspberry PI 4 (4GB RAM) with a BlinkStick USB LED from UAB Tulogic (Lithuania) attached to it. Figure S26: Clamps attached to Insole Unit case. Figure S27: Insole and Crutch Unit in their respective cases. Figure S28: Fully connected insole. Figure S29: Preparation of crutch for acceptance of load cell. Figure S30: Crutch assembly. Figure S31: Crutch Unit attached to shaft. Figure S32: Attaching sleeves to crutch tip. Figure S33: Insole Unit attachement. Table S1: Cost composition of system.

## Abbreviations

The following abbreviations are used in this manuscript:

LLE	Lower-Limb Exoskeleton
IMU	Inertial Measurement Unit
sEMG	Surface Electromyography
FSR	Force-Sensitive Resistor
GRF	Ground Reaction Force
CoP	Center of Pressure
BLE	Bluetooth Low Energy
ADC	Analog-to-Digital Converter
ESP32	A microcontroller series by Espressif Systems
RGB-LED	Red-Green-Blue Light Emitting Diode
RTC	Real-Time-Clock
RMSE	Root Mean Square Error
STD	Standard Deviation
IQR	Inter-quartile Range
PID	Proportional-Integral-Derivative (controller)
