# Supplementary material for: Development and Validation of a Modular Sensor-Based System for Gait Analysis and Control in Lower-Limb Exoskeletons
Source: Sensors (Basel). 2025 Apr 9;25(8):2379. doi: 10.3390/s25082379 (PMC12030982; doi:10.3390/s25082379)
Supplement: Supplementary file 1 [file sensors-25-02379-s001.zip › sensors-3517231-supplementary.pdf]

Supplementary Materials: Replication Guide and Instructions Manual for the Modular Sensory System (MoSeS) for Lower-Limb Exoskeletons

Giorgos Marinou, Ibrahima Kourouma and Katja Mombaur

S1. Introduction

This replication guide is submitted as supplementary material to the paper *Development and Validation of a Modular Sensor-Based System for Gait Analysis and Control in Lower-Limb Exoskeletons* and provides step-by-step instructions to build and set up our proposed system. The following sections outline the design and replication of all individual units, hardware reconstruction, 3D-printing of casings and software installations. For the latest version of files for 3D printing and software, please always visit our GitHub repository at https://github.com/ibokou/MoSeS-LLEx.

S2. Sensor Boards Assembly

The Insole Unit and the Crutch Unit feature a modular design, allowing easy replacement of components in case of damage. The components shared by both are shown in Figure S1. Section S7 provides a bill-of-materials along with a cost-breakdown for all components used in the system.

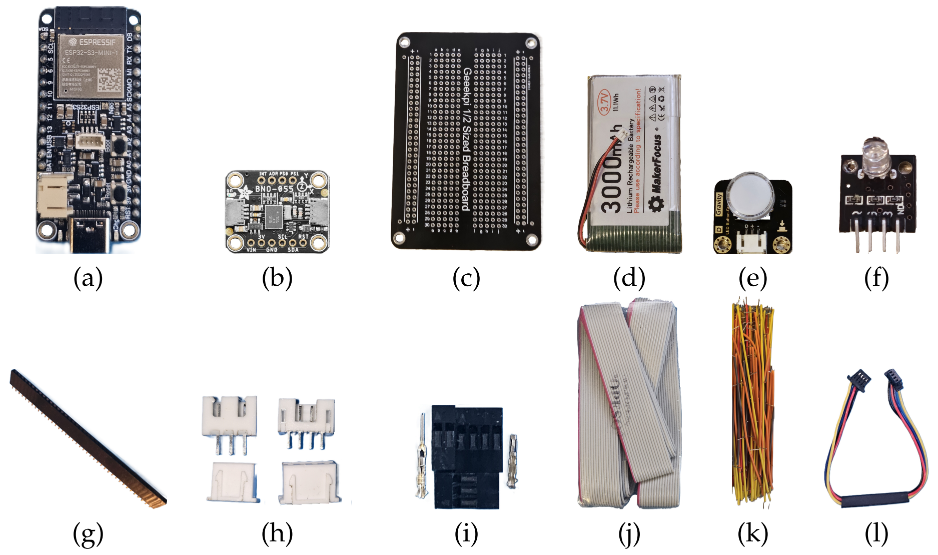

Figure S1. Common components of Insole and Crutch Units. a) Adafruit ESP32-S3 Feather board featuring a STEMMA QT port, enabling easy integration with various sensor boards designed by Adafruit Industries (USA), as well as a JST 2-PH port for the power source. b) Adafruit BNO055 IMU providing a STEMMA QT port. c) Solderable breadboard with 2.54mm hole-to-hole spacing for housing the necessary components. d) Rechargeable 3000 mAh Lithium polymer (LiPo) battery. e) AZ-Delivery KY-016 FZ0455 3-colors RGB LED module for visual signaling. f) DFRobot Gravity LED Button, which goes with a JST to Dupont (female) cable, for turning unit on and off. g) Female pin headers allowing for plug-and-play connections for more expensive components, such as the ESP32, to avoid direct soldering to the board. h) JST-XH 2.54mm connectors for interfacing various components with the breadboard. i) Dupont connectors for interfacing specifically the button cables with the breadboard. j) Ribbon cables, allowing for removable connections between different electrical interfaces. k) Wire bridges for establishing electrical connections between different points on the breadboard. l) STEMMA QT cable for connecting ESP32 and IMU.

S2.1. Practical Information

All components can be easily procured through online platforms, depending on the region and country of purchasing. In case of alternate component choices, please consider the following:

- The Japan Solderless Terminal (JST) connectors can be of a different type as long as the male and female connectors are compatible with each other.
- When choosing a microcontroller unit (MCU) board that provides a battery port (most often JST), make sure that the port matches the connector of the battery cable. Alternatively, existing batteries can be made compatible by replacing the connector.
- More space on the breadboard for the connection between the MCU and the IMU must be planned if no Adafruit products with a STEMMA QT port are used.

S2.2. Crutch Unit board

The manufacturer recommends using the LCM200 load cell in conjunction with the IAA200 amplifier. It has a relatively high demand for input voltage of a minimum of 12.5V which requires the use of a DC-to-DC step-up voltage converter when powered by MCUs, such as the ESP32, which can supply only up to 3.3V with certain pins. These necessary measures to integrate the IAA200 lead to a more bulky and energy-consuming design. Therefore, a compromise has been made to use the HX711 amplifier, eliminating the need for additional components (see Figure S2), as detailed in our paper. When choosing alternative components, the following should be considered:

- The use of the HX711 is highly advised due to its low price, the availability of libraries for software integration and its versatility for use with a wide range of load cells, including industrial grade load cells.
- The use of a different load cell may require a different connector than shown in Figure S2a for interfacing with the breadboard.

Additional components needed to build Crutch Unit board are shown in Figure S2.

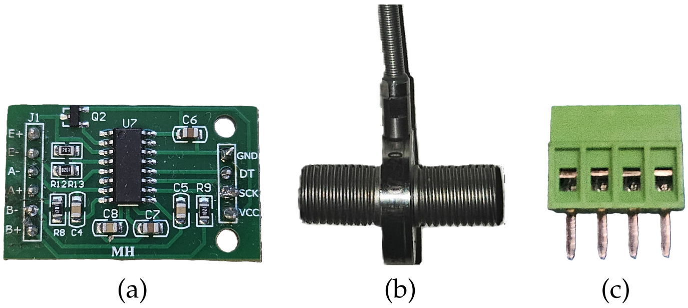

Figure S2. Additional components for Crutch Unit. a) HX711 amplifier. b) LCM200 Futek load cell. c) Screw terminal for interfacing load cell with breadboard.

For assembling the board, please follow the following steps:

1. Create a layout for the placements of all components on the board. Make sure beforehand which pins of the ESP32 need to be exposed for the connection to other components or electrical interfaces. In doing so, consider the default layout of your breadboard as in Figure S3a. Figure S3b shows a possible connection layout for the Crutch Unit. Note that this is from the perspective of the backside, i.e. the pins of the pin headers etc. that need to be soldered are facing you.

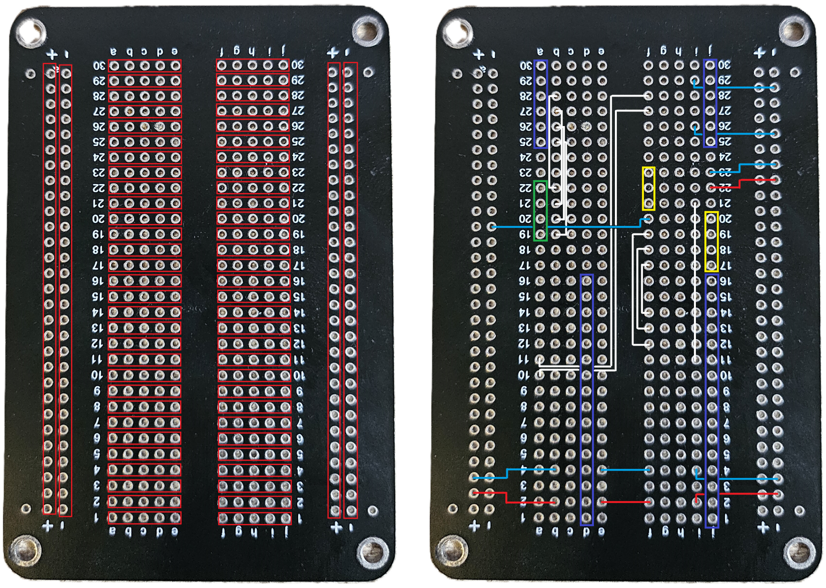

(a) (b)

Figure S3. Breadboard layouts. a) Default layout of the breadboard. Sections or holes that are internally wired together are framed in red. b) Breadboard connection layout for Crutch Unit. Purple frames indicate the location of female pin headers, yellow frames indicate the electrical interfaces for button and LED, which can be, for instance JST ports. The green frame marks the location of the screw terminal. The light blue and red lines represent ground and voltage connections, respectively, and the white lines represent the remaining required wire connections.

2. Solder the pin headers, screw terminals and electrical interfaces for the LED and the button on their respective location. Continue with soldering the wire bridges. The result should mostly resemble what is shown in Figure S4.

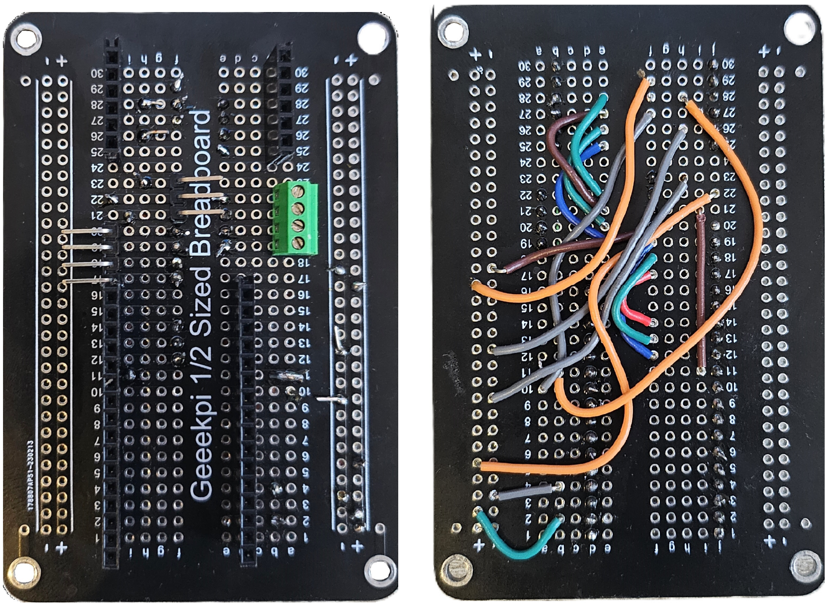

(a) (b)

Figure S4. Front and back of the breadboard with soldered components.

3. Create the cable shown in Figure S5 for the connection between LED and breadboard, by crimping a male JST connector on one side and on the other side a female Dupont connector.

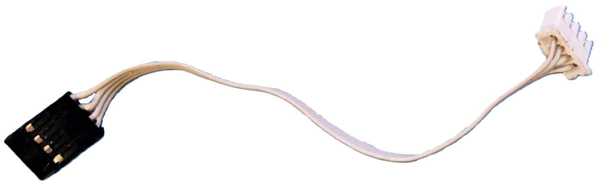

Figure S5. Cable for connection with LED.

4. Create the cable for the connection between button and breadboard if necessary. Crimp a male JST connector on one side and on the other side a male Dupont connector, resulting in the cable shown in Figure S6.

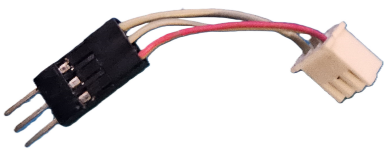

Figure S6. Cable for connection with button.

5. Screw the load cell wires to the screw terminal as illustrated in Figure S7.

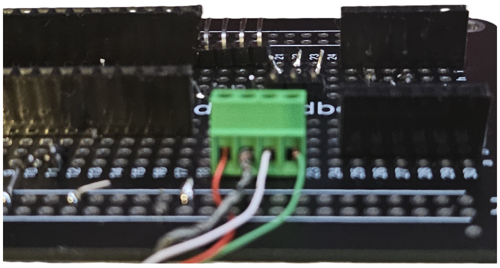

Figure S7. Load cell wires screwed into screw terminal.

6. Plug the ESP32, the HX711 and cables into their designated locations. Make sure that the USB port is facing outwards when plugging the ESP32 to ensure that the pins are connected correctly, following the ones in Figure S8.

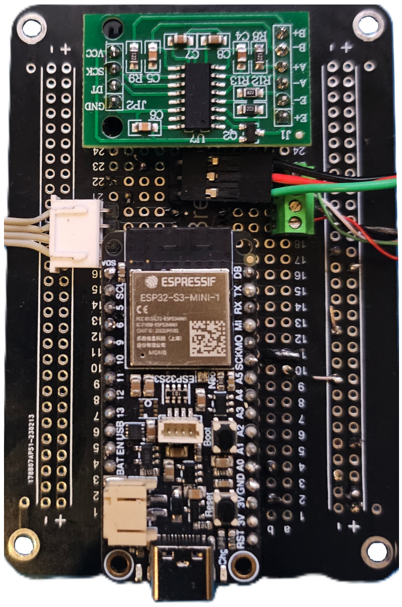

Figure S8. Plugged-in ESP32, HX711 and LED and button cables.

7. Connect the STEMMA QT cable and battery to the ESP32, on the terminals shown in Figure S9.

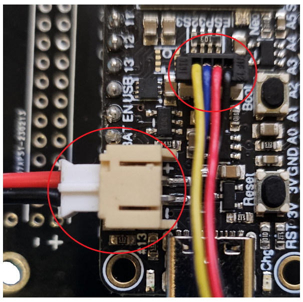

Figure S9. Connected battery and STEMMA QT cable.

S2.3. Insole Unit board

The insole unit board houses the ESP32 and the IMU BNO055, as well as the necessary terminals for connecting to the FSR sensors in the insoles. The ADC onboard the ESP32 is used to read the FSR raw data, with no need for an external ADC. Figure S10 shows the additional components required.

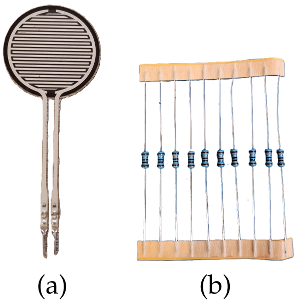

Figure S10. Additional components for Insole Unit. a) force-sensitive resistors (FSR) that saturates at 30kg. b) 10$k\Omega$ resistors.

Please follow the next steps to assemble the insoles board:

1. Similar to the Crutch Unit, first plan the breadboard layout as illustrated in Figure S11.

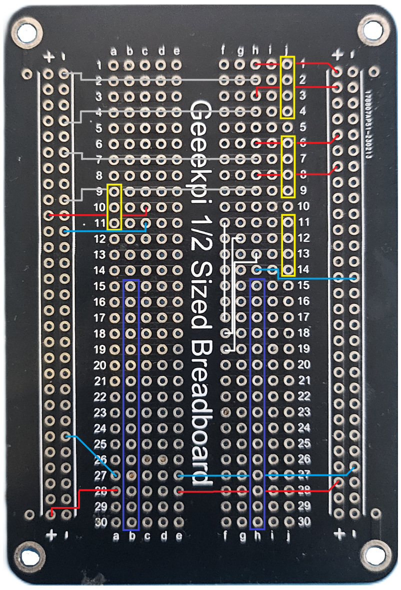

Figure S11. Breadboard connection layout. Same color coding as in S3 with the addition that grey lines represent the resistors.

2. As with the Crutch Unit, start with soldering the pin headers and interfaces. Continue with the resistors and wire bridges, following the wiring in Figure S12.

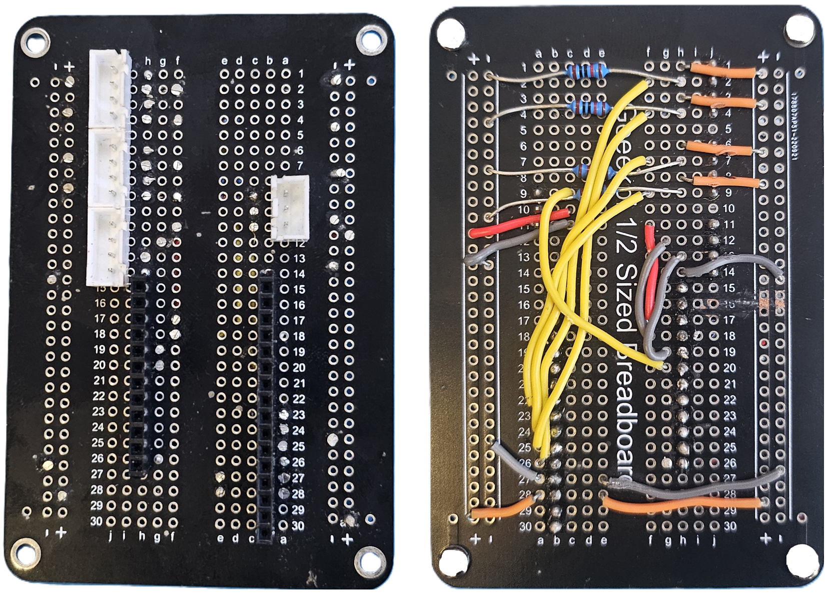

(a) (b)

Figure S12. Front and back of the breadboard with soldered components.

3. Replicate step 3 and 4 in Crutch Unit board.
4. Create the first part of the three-part cable system for the connection between the FSRs placed on the 3D-printed insole and the Insole Unit. Crimp on one side, two male 4-pin JST connectors and on the other side two male 4-pin Dupont connectors, creating the cable assembly shown in Figure S13.

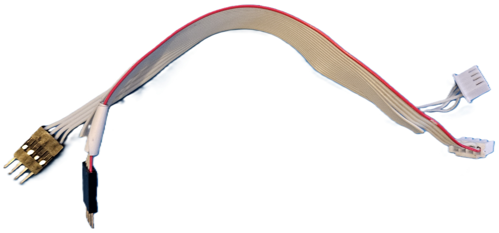

Figure S13. First part of the three-part cable system.

5. For creating the second part of the three-part cable system, crimp on one side two 4-pin female Dupont connectors and on the other side, one 4-pin and one 2-pin female Dupont connector, as shown in Figure S14.

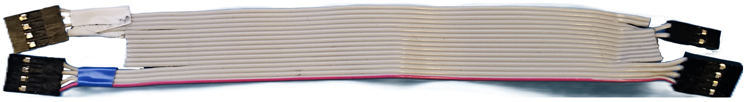

Figure S14. Second part of three-part cable system.

6. Create the third part of the cable system by crimping 2-pin female Dupont connectors on one side and on the other side 2-pin male Dupont connectors (see Figure S15.

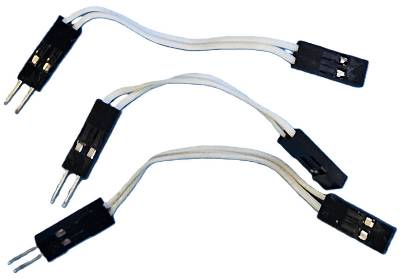

Figure S15. Third part of three-part cable system.

7. Connect the first part of the three-part cable system with the board and replicate steps 6 to 7 in Crutch Unit board, to result with the board shown in Figure S16.

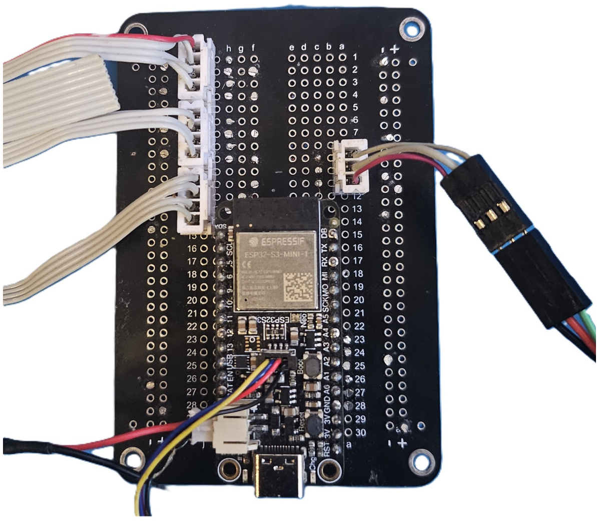

Figure S16. Plugged-in board.

S2.4. Breadboard alignment

It is recommended to use secondary breadboards alongside the breadboards with the components soldered on to ensure an overall compact unit and to allow for space for the LED, button and IMU by aligning both horizontally with the help of spacer sleeves (Figure S17. Consequently, everything fits neatly into the 3D printed cases, while the delicate electrical connections are also protected from physical exposure. M3 is the typical form factor for breadboards or polychlorinated biphenyl (PCB) boards. The steps necessary for this process are explained using the Crutch Unit board, but it is also applicable for the Insole Unit boards.

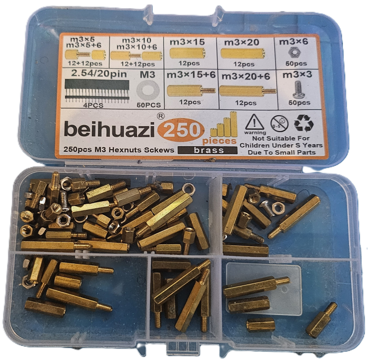

Figure S17. Spacer sleeves package with M3 form factor.

Follow these steps for assembly:

1. Take the breadboard with the circuit soldered on and fixate on each corner a sleeve that is long enough with screws that are inserted from the opposite side, following the markings on Figure S18.

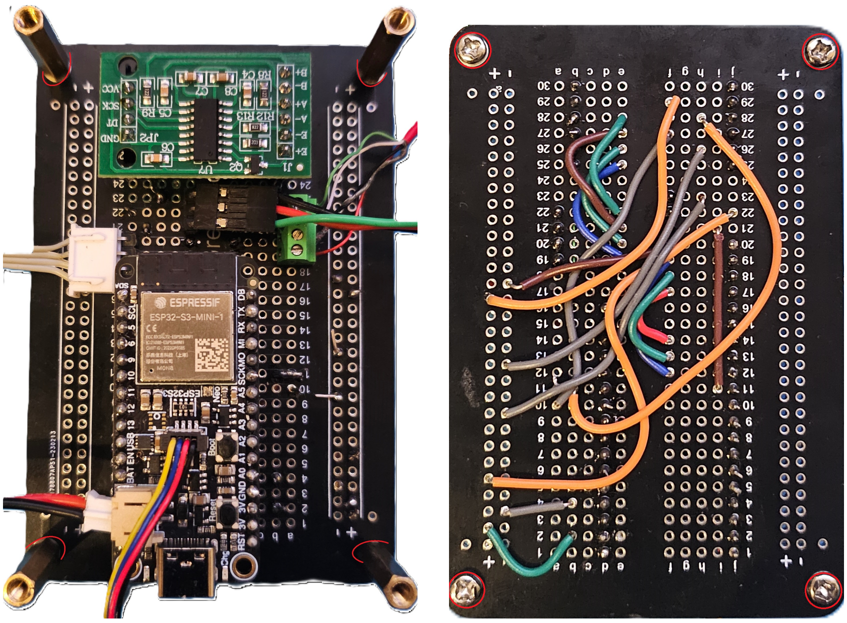

(a) (b)

Figure S18. Breadboard with fixated sleeves on all four corners.

2. Take the secondary breadboard and align it horizontal to the other breadboard. Only fixate it on two corners. The other corner holes are used for attaching the LED and the button, as shown in Figure S19.

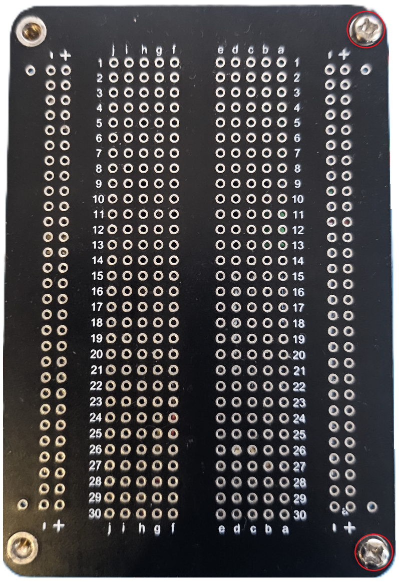

Figure S19. Horizontally aligned breadboards.

3. Fixate the LED and the button on the remaining corners and connect them. Place the battery and the IMU on the remaining space and connect them as well. For ensuring that the IMU remains fixated, solder the pins that come with it beforehand and insert

it into available holes of the breadboard. Use tape if necessary for a more secure fit (see Figure S20).

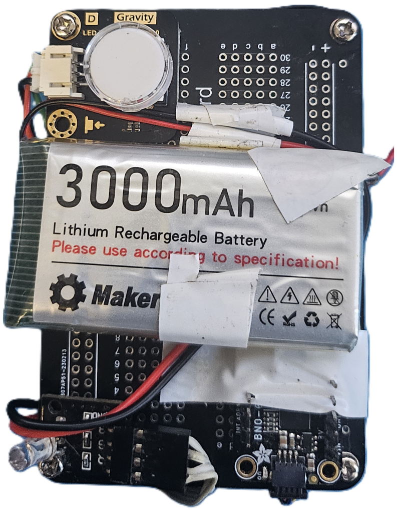

Figure S20. Complete Crutch Unit board.

S2.5. Optional: External Trigger

This system unit is primarily intended to communicate with external systems, such as camera systems, for synchronization of the recording time. Consequently, the effort for building this unit is kept to a minimum as it being most likely stationary there is no need for cases, batteries etc.

For the external trigger, the most important part is the board with a 3.5mm audio jack port, as show in S21b. The assumption is made that the external system allows for a trigger signal being sent via a jack port, which is very common in camera systems. Practically any MCU can be used for the trigger. This guide uses a ESP32 Firebeetle, as can be seen in S21a.

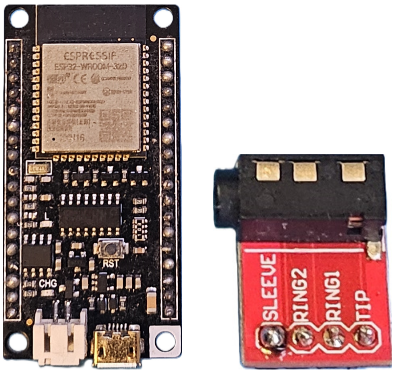

(a) (b)

Figure S21. Components for external trigger. a) ESP32 Firebeetle b) ICQUANZX TRRS 3.5mm audio jack breakout board.

For assembling the trigger, connect the SLEEVE-pin to the ground and the TIP-pin to any digital pin of your MCU by using e.g. Dupont connectors. Make sure that this pin can be set as an output by your MCU in software. Figure S22 shows the final trigger board assembly.

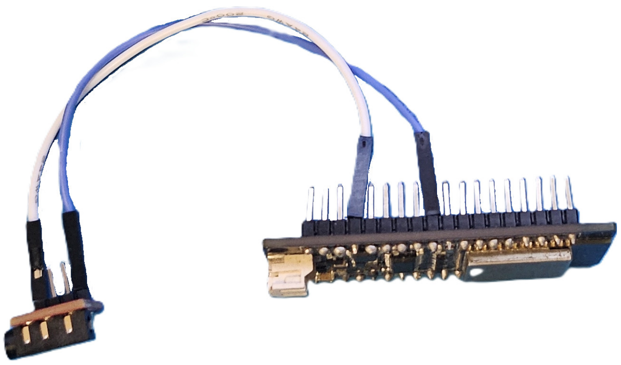

Figure S22. Complete External Trigger Unit board.

S3. 3D Printing

All 3D-printed parts have been designed using AutoDesk's Fusion 360 and sliced using Ultimaker Cura. The Creality Ender 3 S1-Pro was used to print all parts. Any standard FDM 3D Printer equipped with a 0.4m diameter will be able to print these parts.

S3.1. Crutch Unit case

The crutch cases are designed as to fit in the soldered breadboards in the configurations described above. Left and right cases correspond to left and right crutches, and have to be attached accordingly as they ensure the orientation of the IMUs are the same for both. In order to attach the cases on the crutches, two crutch clamps have to be printed per case and used to fasten the cases in between them as shown in Figure S31. Two optional slits are designed on the rim of the cases for passing the load cable through before closing them.

The cases were printed with standard PLA filament (Creality) using Cura's standard preset settings for PLA filament and nozzle size of 0.4 m m. Nozzle temperature was set to 200 degrees Celsius, and bed temperature to 60 degrees Celsius.

S3.2. Hemispherical Sleeve

The hemispherical sleeve provides a semi-elastic interface for the bottom tip of the crutch, and it's provided in order to avoid bending moments about the load cell's central axis. While it's curvature ensures an even distribution of ground reaction forces (GRFs), provides a 'cleaner' contact surface so that the main component of the GRFs aligns as best with the central axis of the crutch. The sleeve should fit most standard rubber t ips. Adding an extra layer of silicon glue can provide extra adhesion for a more secure connection.

The flexible s leeve w as p rinted w ith a 1 .75mm T PU-95A fi lament (C reality) using Cura's standard preset settings for such filaments, a nd n ozzle s ize o f 0 .4 m m . Nozzle temperature was set to 228 degrees Celsius and bed temperature was kept at 0.

S3.3. Insoles

Each insole file contains two parts as the insoles are split in half in order to be able to be printed in a standard 23x23 cm print bed (see Figure S23. The parts can be joined together by a small fit-in-place joint on the bottom of the i nsoles. There are two insoles per size and three sizes (S, M, L) so six insole files in t otal. Printing and snapping the insoles together should be straight-forward.

The flexible insoles were printed with a 1.75mm TPU-90A (NinjaTek NinjaFlex) filament using Cura's standard preset settings for TPU filaments, and a nozzle size of 0.4 mm. Nozzle temperature was set to 237 degrees Celsius and bed temperature to 35 degrees Celsius.

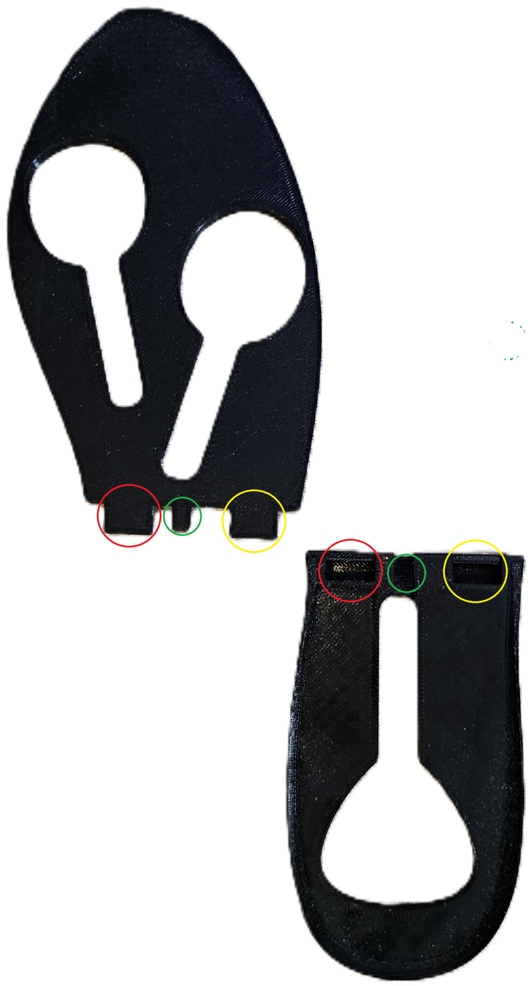

Figure S23. Insole joints.

S3.4. Insole Unit case

The insole cases are designed as to fit in the soldered breadboards in the configurations described above. Left and right cases correspond to left and right crutches, and have to be attached accordingly as they ensure the orientation of the IMUs are the same for both. Two slids are designed in the cases as to allow the ribbon cables from the FSRs to pass through and connect to the terminals. In order to firmly attach the cases to the shoes, shoe clamp files are provided as well. By screwing these clamps on the cases on the locations demonstrated in Figure S26 the clamps can then be adjusted on most standard shoe heels. To further fasten the clamps on the shoe, elastic bands can be used by passing them through the side holes of the clamps and fixing them on the other side's small circular protrusions.

The cases were printed with standard PLA filament (Creality) using Cura's standard preset settings for PLA filament and nozzle size of 0.4 mm. Nozzle temperature was set to 200 degrees Celsius, and bed temperature to 60 degrees Celsius.

S4. Software Installation

All the necessary steps needed for building the different software applications are described in detail in the README files in our Ocean Code capsule.

S4.1. Central Unit

The software for the Central Unit is primarily responsible for initiating the connection with the peripherals once they advertise their service over Bluetooth Low Energy (BLE) and collecting their data. The software assumes that a specific type of USB-LED is used,

as outlined in step 2 in System Integration. Therefore, make sure that it's plugged in or change that part of the code accordingly.

Once the system is turned on, the LED blinks green for a few seconds before switching to blue to indicate that the Central Unit is scanning for the peripherals. If unsuccessful, it enters a restart loop. A steady blue light from the LED signals that all peripherals are connected and that the Central Unit now advertises its service over BLE so that the app in Android Application is able to connect to it.

Once a recording has been started via the app, the Central Unit enters a calibration mode, during which it is necessary that the user remains still to capture precise values. During the calibration mode, the LED blinks yellow.

Shortly before the recording is started, a green steady light is shown and the Central Unit communicates with the external trigger, if one is used. The LED blinks blue during the recording process. When the recording ends, the LED shows a steady green light again, during which the Central unit communicates again with the external trigger, before going back to a steady blue light. During recording, the data is continuously written to a CSV file on the file system.

The app, once completely done, will handle the connection initiation and the calibration as described in Android Application.

For running the software, make sure that a basic Linux operating system (OS) is running on your central unit. The Ocean Code capsule contains two Docker files, one for cross compiling for the Advanced RISC Machines (ARM) architecture, which can be changed to x86 architecture. The other Docker file is for building a Docker image so that the software can run as well in a Docker container.

The software is primarily targeted for a native Linux system. While it is possible to run a Docker container on other operating systems, such as macOS and Windows, containers do not have access to the Bluetooth service on the former and for the latter, some adjustments could be possibly made so that they do have the necessary access. For that, it is necessary that the Docker service uses the Windows Subsystem for Linux (WSL) backend. Furthermore, it needs to be ensured that Bluetooth is activated for the WSL-Kernel, that drivers for the Bluetooth adapters are available or are installed and that the Bluetooth adapter is shared with WSL. For more information, please refer to the WSL project on GitHub.

S4.2. Peripherals

The software of the peripherals allows for the device to advertise a BLE service and transmit data once connected. Crutch Units send IMU and load cell data, while Insole Units send FSR data instead of load cell data. The start process of the peripherals is indicated by slow green blinking, transitioning to slow fast blinking during IMU calibration. Please refer to the documentation of the Bosch BNO055 for the movements required to calibrate it. A service is advertised over BLE to allow for monitoring the calibration state.

After successful calibration, the advertisement switches from calibration state to sensor readings indicated by a steady blue light. A successful connection is indicated by a slow blue blinking LED. Active data transmission is indicated by fast blue blinking.

Any errors during sensor initialization or communication with the ESP32 leads to an immediate restart, a few seconds after a red steady light is shown. Furthermore, peripherals can be brought to sleep mode with a button press of 5 seconds. For the Crutch Units, a button press of one second leads to the tearing of the load cell, which is also teared during startup.

The software is targeted for the ESP32 MCU family. For support of other MCU families, the code needs to be rewritten. The steps for building the software for an ESP32 with a CPU different from the ESP32-S3 are described in the designated README file.

S4.3. Android Application

The Android application specifically tailored to our system is still in development. The Flutter framework is used for the application, which allows for a multi-platform build. The steps required for porting the application to IOS will be described in a designated README file. Our Ocean Code capsule will also contain a workflow document with all the possible interactions with the app. In the meantime, it is possible to interact with the system with publicly available BLE scanner apps, an example of which is shown in Figure S24.

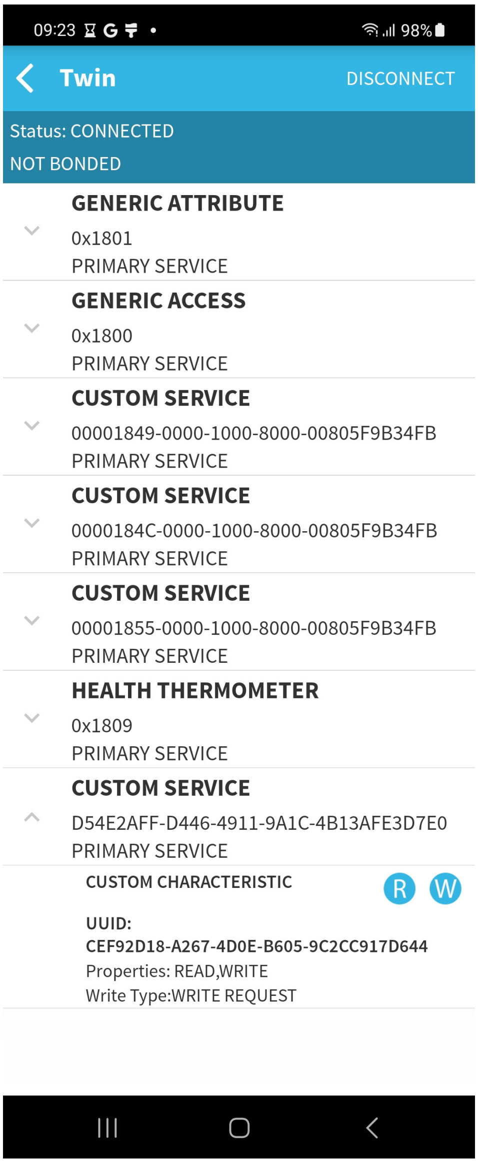

Figure S24. BLE scanner app.

A specific set of commands are accepted by the Central Unit which are sent as a text:

```
start <subject name> <exo|noexo>
stop
restart
shutdown
```

If the Central Unit receives a command that is unknown or in the wrong format, it is blinking red for five seconds, after that it is blinking blue again. If a correct start command with the subject name and mode, meaning exo or noexo, is provided than the Central Unit starts the recording of data. A correct start command looks like:

```
start subject1 noexo
start subject2 exo
```

S4.4. Optional: External trigger

The software is entirely written in Arduino, so any MCU platform that supports Arduino, can run the software right away. Only the output pin needs to be adjusted in the code. Once turned on, the external trigger advertises a read service over BLE. The Central Unit access this service, when a recording is stopped or started. The output pin is set to high for a specific amount of time. Please refer to the documentation of your external system regarding duration of the trigger signal.

S5. System Integration

If you followed this guide so far, you should have the following:

- Fully functional sensor boards for Crutch and Insole Unit
- Cases and clamps for the Crutch, Insole and Central Unit, as well as sleeves for the crutch.

To bring everything together, follow these steps:

1. Connect your ESP32 boards with your PC and build and upload the software. Make sure that the correct applications are uploaded to the boards (left vs right).
2. Prepare the board PC for the Central Unit by uploading the designated software to it. Pay attention to the instructions in Bill-of-Materials. Plugin the USB-LED as shown in Figure S25.

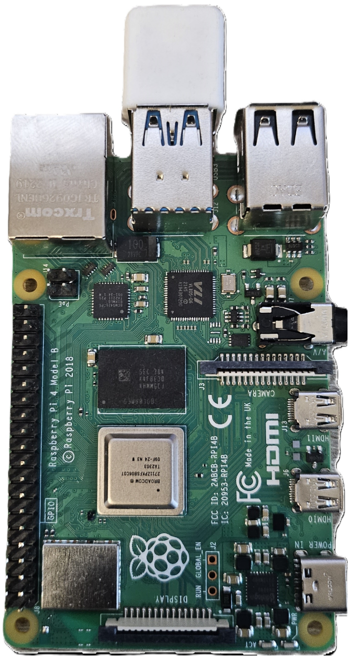

Figure S25. Raspberry PI 4 (4GB RAM) with a BlinkStick USB LED from UAB Tulogic (Lithuania) attached to it.

3. Prepare the case for the Insole Unit. Align the clamps with the back of the case (see Figure S26). Fixate them with screws and bolt nuts. The cases for the Crutch Unit are ready to be used directly.

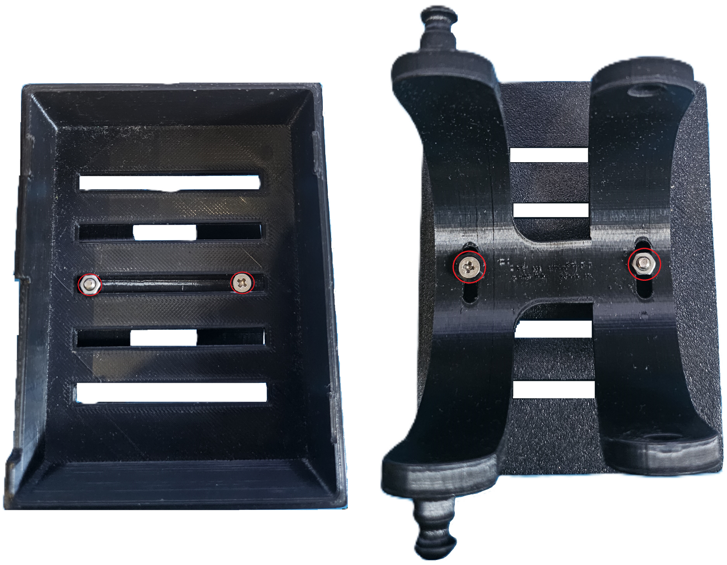

(a) (b)

Figure S26. Clamps attached to Insole Unit case.

4. Put the boards into their cases, make sure that the cables are exposed correctly, corresponding to each side of the insole or crutch, shown in Figure S27.

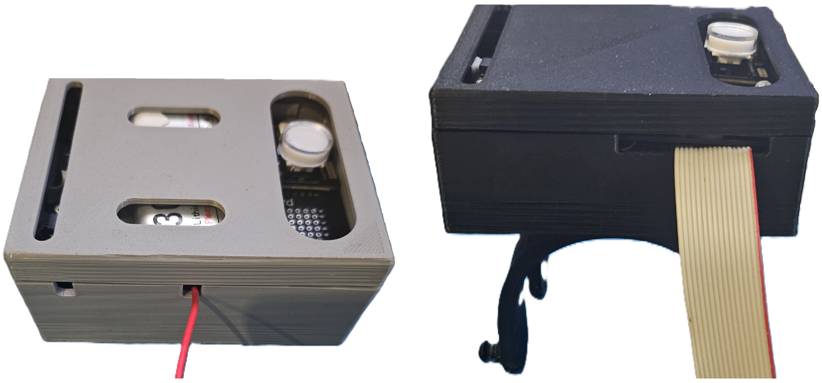

(a) (b)

Figure S27. Insole and Crutch Unit in their respective cases.

5. Prepare the insoles that are worn in the shoes by fixating the FSRs on the designated imprints. Connect the third part of the three-part cable system to the FSRs then the

second part to the third part followed by connecting the first part which results in what is shown in Figure S28.

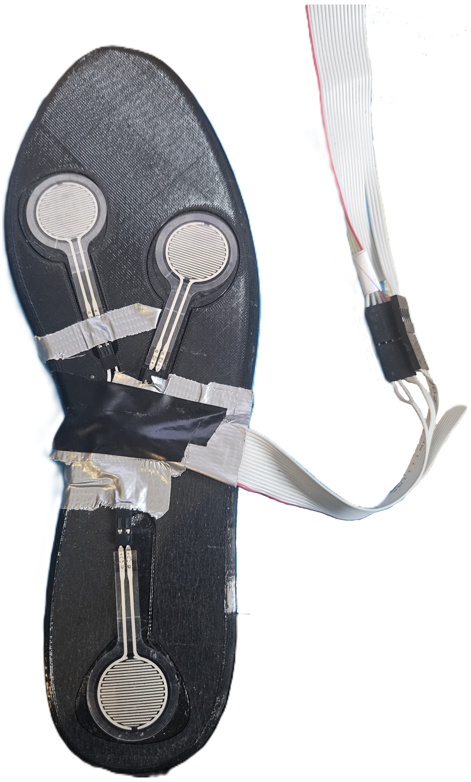

Figure S28. Fully connected insole.

6. The preparation of the crutch for acceptance of the load cell begins by cutting the crutch shaft just above the rubber tip along the cross-sectional area. Insert an aluminum rod whose outer diameter matches the inner diameter of the crutch shaft, ensuring a tight fit with near-zero tolerance. Repeat this process for the crutch tip as well. Finally, drill both the aluminum-fitted shaft and tip with a drill head whose size corresponds to the that of the load cell. In case of the LCM200 it must be the 3/8-24-Thread form factor. Figure S29 indicates the aluminium rod inserted in the drilled crutch, both at the upper cylinder (part a) and bottom tip (part b).

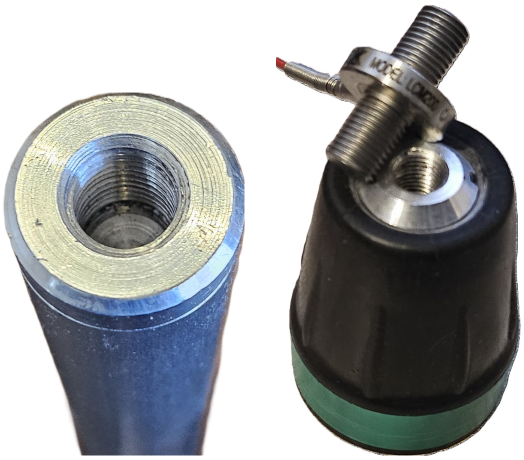

(a) (b)

Figure S29. Preparation of crutch for acceptance of load cell. a) Shaft with drilled hole b) Crutch tip with drilled hole.

7. Screw the load cell to the shaft and the crutch tip, following Figure S30.

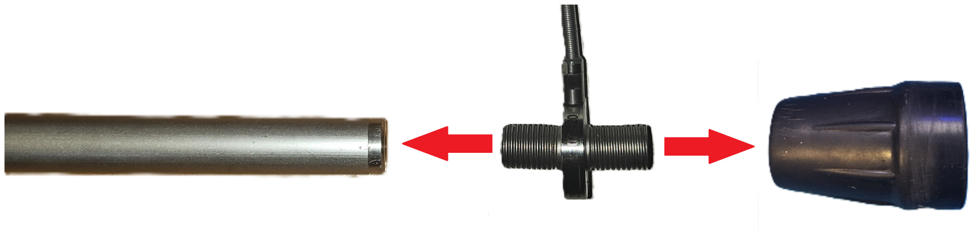

Figure S30. Crutch assembly.

8. Attach the Crutch Units to your crutch by first taking a clamp and inserting it to the shaft (see Figure S31). Then on top of it place your Unit and then the clamp. Optionally, use tape to make everything more secure.

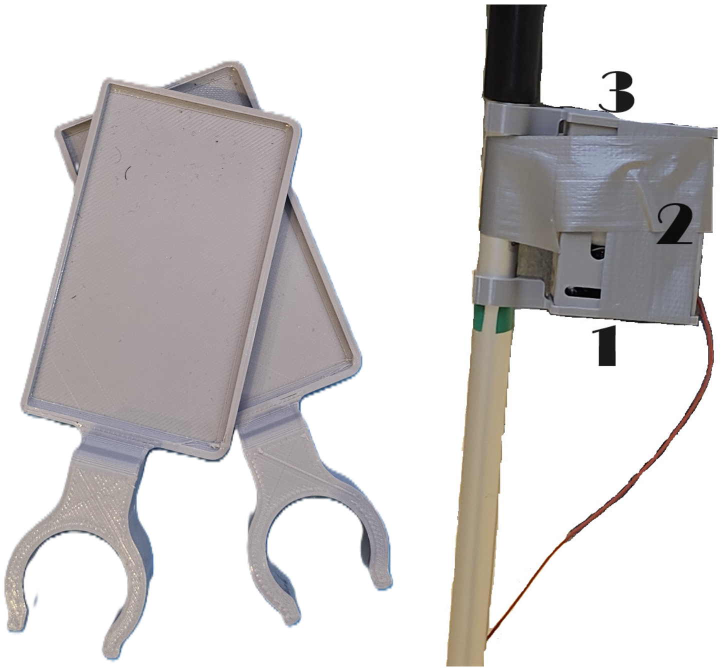

(a) (b)

Figure S31. Crutch Unit attached to shaft.

9. Attach the sleeves to the crutches tip as shown in Figure S32. It is highly advised to not skip this step, since the sleeves allow for offloading shear forces applied by the user, thus preventing potential damage to the load cells.

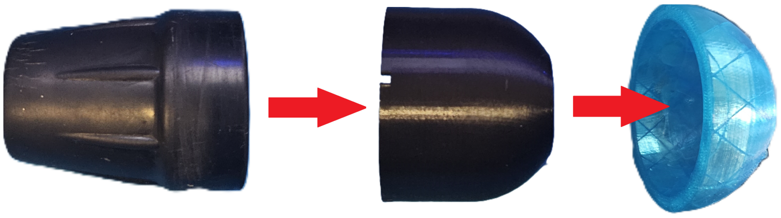

Figure S32. Attaching sleeves to crutch tip.

10. For wearing the Insole Unit, it is advised to do that in a seated position. Put the insoles into the shoes and wear them. Ask a second person to attach the Unit right above your ankle. Use rubber bands in the front that are spanned from knob to knob for a secure attachment to the user, following Figure S33.

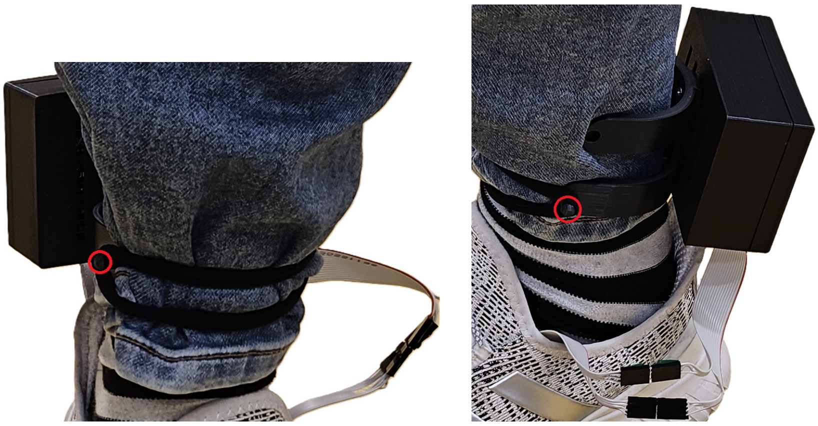

(a) (b)

Figure S33. Insole Unit attachement.

11. Attach the Central unit case if possible to your exoskeleton and insert into them your unit and your power source. Alternatively, if your range of operation is small, it is also possible to leave the Central Unit stationary.

S6. System Operation

After the peripherals and the Central unit have been turned on, please refer to Android Application for how to operate and communicate with the system.

S7. Bill-of-Materials

Table S1 provides a breakdown of costs for all components outlined above, required to design the system. It includes costs for 3D-printing, assembly materials, as well as all sensors used. The grand total for the system is indicated in the bottom.

Expenses	Unit price	Amount	Total Price
Rasberry PI 4 Model B (4 GB)		1	€68.32
BlinkStick USB LED		1	€16.49
SD card (16 GB)		1	€5.37
ESP32 S3 Feather	€23.90	4	€95.60
LiPo battery (3000 mAh)		4	€32.99
BNO055 IMU	€35.64	4	€142.56
STEMMA QT cable	€3	4	€12
Solderable Bread boards		8	€23.98
DFRobot Gravity LED Button	€5.18	4	€20.72
3-colors RGB LED	€1.30	4	€5.20
HX711 amplifier	€1.90	2	€3.80
Fukek LCM200 load cell	€480	2	€960
FSR	€9.09	6	€54.54
Pair of crutches		1	€16.06
3D printing			€20
Assembly material (wires, etc.)			€4
Grand total			€1481.63

Table S1. Cost composition of system.

S8. Troubleshooting

The following provides troubleshooting advice for issues that may occur while assembling or operating the system.

S8.1. Issue: Frequent connection loss between Central Unit and peripherals

Try to adjust the connection interval for the individual connections. In an environment with various other Bluetooth devices, a low connection interval may increase the risk of interference.

S8.2. Issue: Diminishing data throughput, the higher the count of devices connected to the central unit

Usually, a Bluetooth adapter cannot maintain the throughput it delivers in single client mode if multiple peripherals are connected. One solution is to increase the number of Bluetooth adapters for the Central Unit. Its code is written, such that all available adapters are detected and the peripherals are distributed among them.

If possible, a dedicated adapter is selected automatically which is used for the connection with the app and for the external trigger, since for both a high throughput is not needed. Decreasing the connection interval also leads to a higher throughput, but with the risk of more frequent connection losses, as mentioned before.

Apart from that, the type of the adapter and the supported Maximum Transmission Unit (MTU) size plays also a crucial role in the total throughput of the system. The MTU determines how much byte of data can be transferred in a single data packet transmission. If the data is too large, then the transmission must fragment the data into smaller packets, which negatively impacts the throughput.

Finally, there are much more parameters that can be configured to improve the connection capabilities. For that, please refer to the official Bluetooth manuals.

S9. Contact

For further questions, information and additional materials please contact one of the two authors:

Giorgos Marinou: gdmarinou1@gmail.com

Ibrahima Kourouma: him.kourouma@hotmail.com,

and visit our repository for downloading our open-source software code, STL files and the latest updates (including our new custom Android App) at https://github.com/ibokou/MoSeS-LLEx.
